# Anticipatory self-efficacy predicts live musical performance: development and validation of the Music Aptitude Self-Efficacy Scale

**DOI:** 10.3389/fpsyg.2026.1869088

**Published:** 2026-06-19

**Authors:** Gulce Coskun Senturk, Asli Kaya, Cagri Basbug

**Affiliations:** Department of Fine Arts Education, Faculty of Education, Mugla Sitki Kocman University, Mugla, Türkiye

**Keywords:** adolescence, behavioral assessment, musical aptitude, performance anxiety, psychological resilience, psychometrics, self-efficacy

## Abstract

High-stakes musical aptitude examinations represent a significant source of evaluative stress for adolescent musicians, yet the psychological mechanisms that influence success in these environments require further empirical investigation. Grounded in social cognitive theory, this study developed and psychometrically evaluated the Music Aptitude Self-Efficacy Scale (MASES) to assess the multidimensional anticipatory beliefs of adolescents navigating these examinations. Utilizing a large adolescent sample (*N* = 2030), exploratory and confirmatory factor analyses supported a triadic structure comprising Cognitive-Auditory, Psychomotor-Performance, and Affective Regulation dimensions. The scale demonstrated internal consistency and concurrent validity against both domain-general and music-specific self-efficacy measures. Furthermore, predictive validity was examined through a live performance simulation evaluated by an expert jury, revealing a positive correlation between candidates’ pre-examination self-efficacy scores and their objective technical performance under stress. These findings indicate that the scale serves as a psychometric instrument for evaluating performance-related self-efficacy beliefs. By providing insights into adolescents’ perceived competence prior to evaluation, this tool can assist music educators and counselors in understanding students’ preparatory needs, contributing to a supportive educational framework.

## 1 Introduction

Music is not just entertainment; it is a complex auditory stimulus that activates various cognitive processing systems (Sust et al., 2026). Music education is a multidimensional and dynamic process requiring the simultaneous development of an individual’s cognitive, affective, and psychomotor skills (Hallam, 2010). Identifying particularly talented individuals and guiding them toward professional music education understanding their self-perception in music is critical for music educators (Strauss et al., 2023; Tunca et al., 2024; Blasco-Magraner et al., 2025). Although talent is considered an innate potential, transforming this potential into performance is directly related to the individual’s motivation, anxiety management, and, most importantly, their belief in their capacity (McPherson and McCormick, 2006). Performance anxiety (triggering stressor) requires candidates to utilize coping and emotion regulation (active strategies) skills. Successful use of these strategies provides resilience during performance and contributes to the student’s psychological well-being in the long term.

To precisely operationalize musical anticipatory self-efficacy, the MASES integrates four foundational frameworks into a cohesive, unified construct. In this integrated model, Bandura’s social cognitive theory serves as the overarching psychological mechanism, representing the core ‘belief’ in one’s capabilities. However, because high-stakes musical performance is inherently multidimensional, this generalized belief must be mapped onto specific performance domains. Therefore, we integrated Bloom’s cognitive taxonomy—specifically adapted for the musical context through Gordon’s concept of ‘audiation’ (the cognitive foundation of internalizing and comprehending sound)—to construct the Cognitive-Auditory dimension. Concurrently, Simpson’s psychomotor taxonomy was utilized to operationalize the physical execution and mechanical mastery required in instrumental performance, forming the Psychomotor-Performance dimension. Finally, the affective domain of Bloom’s taxonomy was incorporated to capture the emotional regulation necessary to manage pre-performance anxiety. Thus, rather than being an eclectic aggregation, the MASES represents a structured, operational synthesis: Bandura’s self-efficacy acts as the central psychological engine, systematically driving the cognitive (Gordon/Bloom), psychomotor (Simpson), and affective domains of musical aptitude.

### 1.1 The nature and psychological dimension of aptitude tests

Aptitude test scores are generally interpreted similarly for candidates with the same overall score. However, research has found evidence of differences among candidates in strategies and the consistent application of proper procedures during testing, and it is clear that each student, despite having different strategies and skill levels, is taking those exams for the same purpose (Sideridis and Alahmadi, 2022; Candia et al., 2023; Christiner et al., 2023; Hambrick et al., 2023; Hauenstein et al., 2024). Within these highly demanding educational environments, the severe academic stress associated with high-stakes testing poses a significant risk to the emotional health and psychological well-being of adolescents. Therefore, the capacity to implement effective coping strategies is vital not only for academic success but also for safeguarding student well-being. Additionally, talent exams, the most important threshold for transitioning to professional music education, are challenging processes that test not only candidates’ musical auditory and performance skills but also their capacity to perform under high stress. Although there are numerous studies in the literature on musical performance anxiety (Kenny, 2011; Braden et al., 2015; Burin and Osório, 2016; Blume et al., 2017; Brooker, 2018; Barros et al., 2022; Bakhtiari et al., 2025) and general academic self-efficacy, the limited number of measurement tools specifically focusing on the “aptitude test process” is noteworthy. However, in a candidate’s success in the aptitude test, their self-efficacy belief in their aptitude is as influential as the aptitude itself.

### 1.2 The Türkiye context: music aptitude tests for transition to higher education

In Türkiye, admission to Music Education Departments within Faculties of Education, which provide professional music education and form the foundation of the music teacher training process, is generally carried out through multi-stage “Special Talent Exams” (auditory perception, sight-reading, instrumental performance, singing, etc.) organized by universities, attended by high school graduates (16–18 years old) and young adults who have graduated from high school (Güdek, 2013). These highly competitive entrance exams for higher education expect candidates to simultaneously demonstrate quick reflexes, technical proficiency, and theoretical knowledge in front of an expert jury. However, the current examination evaluation system focuses solely on the candidate’s musical ability, while excluding “aptitude test self-efficacy,” which determines the capacity to demonstrate this ability under intense pressure (Ryan and Andrews, 2009; Papageorgi et al., 2013). This situation can lead to candidates, despite possessing high musical potential and technical skills, being excluded from the system due to a decline in their performance in front of the jury caused by low self-efficacy. This systemic exclusion fundamentally contradicts the principles of sustainable, inclusive, and equitable quality education (SDG 4) (Sáiz-Manzanares et al., 2020; Castellanos et al., 2021; Yang et al., 2025). Losing talented individuals simply due to unmanaged academic anxiety rather than a lack of actual musical capacity results in an unsustainable waste of human potential. Therefore, addressing this gap by evaluating candidates’ coping strategies is essential for creating a fairer talent selection ecosystem (Trigueros et al., 2020).

### 1.3 Conceptual model: the multidimensional structure of music aptitude self-efficacy

**Figure 1:**
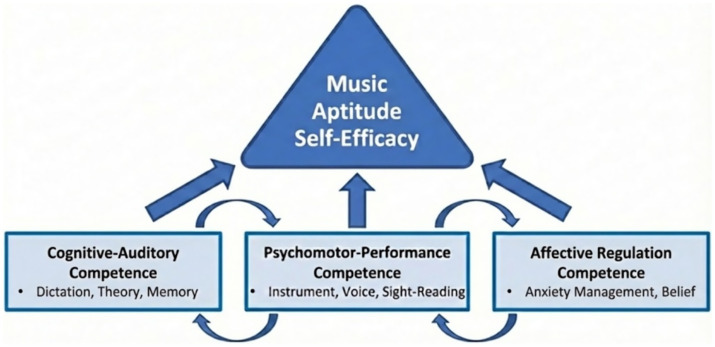
Music aptitude self-efficacy triadic model.

In this study, self-efficacy perception regarding music aptitude tests was modeled not as a one-dimensional structure, but as a dynamic interaction of cognitive, psychomotor, and affective processes. The three-factor structure obtained from Exploratory Factor Analysis (EFA) was conceptualized within the framework of the Music Aptitude Self-Efficacy Triadic Model, which aligns with fundamental theories in educational psychology (see Figure 1).

The model is constructed from a synthesis of Cognitive (Gordon/Audiation), Psychomotor (Simpson/Execution), and Affective (Bandura/Anxiety Management) theories. The arrows represent the dynamic interaction and reciprocal reinforcement between the dimensions. This model is based on a synthesis of Bandura’s (1986) Social Cognitive Theory and Bloom et al.’s (1956) taxonomy of educational goals (Cognitive, Affective, Psychomotor) in the context of music education. The components of the model are detailed below.

#### 1.3.1 Cognitive-auditory efficacy

The first dimension of the model encompasses the processes of mentally processing, encoding, and memorizing musical data. This dimension is based on Gordon’s (2007) “Audiation” theory. The candidate’s ability to write melodic dictation, recognize intervals, and master music theory is related to Bandura’s “Cognitive Readiness” as a source of self-efficacy (Bandura, 1997). This high level of self-efficacy reflects the presence of mental schemas necessary for the candidate to manage uncertainty during the aptitude exam (McPherson and McCormick, 2006).

#### 1.3.2 Psychomotor-performance efficacy

The second dimension involves the transformation of musical thought into physical action, such as vocal or instrumental performance. This dimension is related to the com-plex overt response level in Simpson’s (1966) psychomotor domain taxonomy. The candidate’s mastery of the instrument, belief in overcoming technical difficulties (seeing, etc.), and bodily control are based on Bandura’s (1997) “Experiences of Mastery”. The belief in one’s technical competence in musical performance is the strongest predictor of minimizing the fear of making mistakes during an exam (Hallam, 2010).

#### 1.3.3 Affective regulation and readiness

The third dimension encompasses the candidate’s ability to manage test anxiety, motivational orientation, and perception of social support. This dimension is directly related to Bandura’s (1997) principle of “Physiological and Affective States” influencing self-efficacy. The ability to cope with performance anxiety and the belief in one’s ability represent the “valuing” and “organizing” levels of the affective domain as defined by Krathwohl et al. (1964). Ritchie and Williamon (2012) emphasize that high affective self-efficacy increases resilience in musical performance. In the context of educational psychology, this affective dimension operationalizes the coping strategies necessary for adolescents to navigate intense academic stressors, directly reflecting their capacity to preserve their psychological well-being (SDG 3) under evaluative pressure.

### 1.4 Gaps in the literature and the importance of research

A review of the literature on music education and performance reveals that the decisive role of self-efficacy and motivational processes in performance success has been strongly demonstrated by McPherson and McCormick (2006) and Ritchie and Williamon (2012). However, these studies, which focus on students currently receiving conservatory training, are limited in their ability to measure the “high-stakes” exam psychology experienced by candidates at the entry stage of the system, and the specific cognitive requirements of these exams, such as auditory (melodic dictation/theory) and instantaneous deciphering, using a holistic model. Similarly, the measurement tools developed by Zelenak (2015, 2024) focus more on determining general class participation and motivational orientations, while not offering a diagnostic framework for predicting the outcome of talent exams. On the other hand, the most widely used Kenny (2011) Music Performance Anxiety Inventory (K-MPAI) addresses the performance process from a negative perspective, viewing “anxiety” as a pathological condition. However, current positive psychology approaches argue that success is explained not only by the “absence of anxiety” but also by the “presence of belief in one’s ability (self-efficacy)”. In this context, while there is a vast literature on musical self-efficacy and musical performance anxiety, the MASES (Music Aptitude Self-Efficacy Scale) fill a significant methodological and theoretical gap in the literature as it is the measurement tool to address the exam process not solely in terms of performance or anxiety, but within the framework of the triadic model, built upon cognitive, psychomotor, and affective pillars.

### 1.5 Current study and hypotheses

The main originality of this study lies in its specific application of these structures to the context of high-stress music aptitude examinations and its presentation of a triadic model encompassing cognitive-auditory, psychomotor-performance, and affective regulation components. Based on the theoretical framework and the identified gaps in the literature, the primary aim of the current study is to develop and validate the Music Aptitude Self-Efficacy Scale (MASES) for adolescent musicians preparing for high-stakes aptitude exams. Rather than approaching performance anxiety as solely a pathological issue, this study models exam-specific self-efficacy as a dynamic Triadic Model consisting of cognitive, psycho-motor, and affective competencies.

To validate this model and establish the psychometric properties of MASES, the following hypotheses were formulated:

*H1* (Structural Validity): The MASES will exhibit a robust and valid multidimensional structure corresponding to the proposed Triadic Model, specifically comprising Cognitive-Auditory, Psychomotor-Performance, and Affective Regulation dimensions.

*H2* (Dual-Criterion Concurrent Validity): The MASES total score and its sub-dimensions will demonstrate theoretically consistent, statistically significant positive correlations with both domain-general self-efficacy measures (the SEQ-C) and domain-specific musical beliefs (the SESRMA).

*H3* (Predictive Validity): The pre-examination MASES scores of the candidates will significantly and positively predict their objective, real-time performance scores as evaluated by an independent expert jury within a high-stakes, real-world aptitude exam simulation.

## 2 Materials and methods

### 2.1 Research design

This research is a methodological study aiming to develop and validate the Music Aptitude Self-Efficacy Scale (MASES), a specialized measurement tool designed to assess the self-efficacy perceptions and psychological resilience of adolescent musicians preparing for music aptitude exams. To systematically develop the scale, an Exploratory Sequential Mixed Methods Design was adopted (Creswell and Clark, 2007). The research was executed in two main phases.

In the initial qualitative and developmental phase, a comprehensive literature review was conducted to define the theoretical construct. Subsequently, an item pool was generated, and content validity was established based on expert evaluations utilizing the Davis Technique (Davis, 1992). The face validity and comprehensibility of the draft scale were then refined through cognitive interviews with the target population using think-aloud protocols.

In the second quantitative phase, robust psychometric analyses were performed on a large-scale cross-sectional dataset (*N* = 2,030). This phase included Exploratory and Confirmatory Factor Analyses (EFA and CFA), and comprehensive reliability assessments (Cronbach’s *α* and McDonald’s *ω*). Furthermore, the ecological and predictive validity of the scale were rigorously tested through a real-world stressor simulation involving a subsample of 50 candidates evaluated by a 5-person live expert jury, while convergent validity was established via comparisons with existing self-efficacy instruments.

The research was conducted in strict accordance with the Helsinki Declaration. Ethical approval was obtained from the Mugla Sitki Kocman University Human Research Ethics Committee (Date: 28.04.2021, Decision No: 210190/162). Before data collection, written informed consent was obtained from all participants and, for those under 18, from their parents or legal guardians. Separate permissions were also obtained from the Ministry of National Education due to the research being conducted in schools. Due to the use of an analysis method not included in the planning phase of the research, the addition of a researcher specializing in analysis to the research team with strong support, and a change in the research title, a second ethical committee approval was obtained from the Mugla Sitki Kocman University Educational Sciences Research Ethics Committee before the publication phase (Date: 09.04.2026, Decision No: 260086/85).

### 2.2 Participants

The target population for this study comprised 11th and 12th grade students attending the music departments of Fine Arts High Schools, specifically those aiming to take national or international high-stakes music aptitude exams. A purposive sampling strategy was employed in accordance with the principles of accessibility and suitability for the re-search objectives.

Initially, data were collected from a total of 2,165 participants. Following a rigorous data screening and cleaning process, the final analyses were conducted on a robust sample of 2,030 participants. To thoroughly test the construct validity of the scale and ensure the cross-validity of the model, the “Split-Sample Method” recommended in the psychometric literature was applied (Anderson and Gerbing, 1988). Accordingly, the dataset was randomly partitioned into two independent subgroups for factor analyses, alongside holistic evaluations for validity. The sample details are as follows:

Group 1 (EFA group): Used for Exploratory Factor Analysis, this subset consisted of 997 participants (M age = 17.23, SD = 0.66; 49.4% female, 49.2% male, 1.4% who identified with another gender category; 43.6% 11th grade, 56.4% 12th grade).

Group 2 (CFA group): Used for Confirmatory Factor Analysis, this independent subset consisted of 1,033 participants (M age = 17.23, SD = 0.63; 51.5% female, 47.2% male, 1.3% who identified with another gender category; 41.7% 11th grade, 58.3% 12th grade).

Group 3 (Validity group): Used for convergent and criterion-related validity analyses, representing the merged total sample of 2,030 participants (M age = 17.23, SD = 0.64; 50.5% female, 48.2% male, 1.3% who identified with another gender category; 42.7% 11th grade, 57.3% 12th grade).

Group 4 (Predictive validity group): A nested subsample of 50 candidates (M age = 18.12, SD = 0.39; 44.0% female, 56.0% male; 100.0% 12th grade) who participated in a real-world predictive validity simulation evaluated by an expert jury.

The sample size of this study vastly exceeds the minimum criteria suggested in the literature. According to Comrey and Lee (2013), sample sizes approaching or exceeding 1,000 are classified as “excellent.” Furthermore, Tabachnick and Fidell (2019) suggest a minimum observation-to-item ratio of 5:1 or 10:1. With 35 initial items, both the EFA (*n* = 997) and CFA (*n* = 1,033) groups provide an observation-to-item ratio of approximately 28:1, indicating exceptional statistical power and sampling adequacy for multivariate analyses.

### 2.3 Procedure

To create the item pool of the scale and ensure its content validity, the Davis Technique, which is accepted in the literature as a systematic consensus method, was used (Davis, 1992). This process allowed experts to express their opinions independently and anonymously, thus avoiding group effect. The scale development process was carried out by following the standard procedures suggested by DeVellis and Thorpe (2021):

#### 2.3.1 Item generation

The following processes were carried out during the item pooling phase:

Participants and Sampling: To create the item pool for the scale and to test the cultural suitability of the theoretical structure, criterion sampling from purposive sampling methods was used (Patton, 2014). The study group consisted of 10 participants who were either preparing for or had experience with national or international music aptitude exams. To ensure a heterogeneous group structure, participants were selected from among graduates who had completed their exam experience (*n* = 3), 12th-grade students in their exam year (*n* = 4), and 11th-grade students in the preparation process (*n* = 3) (Table 1).

**Table 1:** The profile of study group (Qualitative phase).

Session	Code	Gender	Grade	Instrument	Musical background (year)	Exam experience
1st	P1	F	11.	Piano	4	Preparation
	P2	M	12.	Violin	6	Preparation
	P3	F	Graduated	Baglama	3	3 times
	P4	M	12.	Guitar	4	Preparation
	P5	M	12.	Flute	4	Preparation
2nd	P6	M	11.	Piano	3	Preparation
	P7	F	12.	Viola	6	Preparation
	P8	F	11.	Guitar	5	Preparation
	P9	M	Graduated	Cello	6	2 times
	P10	F	Graduated	Violin	8	4 times

Data Collection Process: In data collection, the “Semi-Structured Focus Group Inter-view” technique was preferred, allowing participants to interact with each other and generate in-depth data (Krueger and Casey, 2015). Since the number of participants (*N* = 10) exceeded the ideal group size suggested in the literature and could reduce interaction, the interviews were conducted in two separate sessions of 5 people each (Morgan, 1997). Each session was conducted outside of class time in a quiet and distraction-free environment in the research school building after obtaining parental consent, and lasted an average of 50–60 min. During the interviews, the researcher acted as the moderator, while an assistant researcher observed and noted nonverbal responses during the process.

Recording and Transcription: To prevent data loss and ensure the reliability of the analysis, all interviews were recorded using an audio recorder after obtaining “Informed Consent Forms” from the participants. During the interviews, the researcher also handwritten field notes containing keywords and key points (Creswell, 2013). Following the interviews, the audio recordings were transcribed verbatim without any corrections, resulting in a raw dataset of 24 pages.

Interview Protocol: In the interviews, a “Semi-Structured Interview Form” based on the theories of Bandura, Gordon, and Simpson was used. The questions were structured from general to specific to reveal the candidates’ perceptions of the cognitive, psychomotor, and affective dimensions of the aptitude test. In addition, probe questions were used to deepen the participants’ responses (Yıldırım and Simsek, 1999). The main questions and probe questions asked were as follows:

Cognitive-Auditory Dimension (Focused Gordon): Main question; “When considering the auditory stage of aptitude tests, what are the moments that challenge you the most or make you think, ‘I can’t do it’?”. Example probe question; “What kind of confusion do you experience in your mind, especially when dictating or hearing intervals?”

Psychomotor-Performance Dimension (Focused Simpson): Main question; ““When playing your instrument or singing, what are your thoughts on technical difficulties that might affect your performance in the aptitude test environment? “. Example probe question; “What do you feel in your body when asked to sight-read a piece you do not know?”

Affective Regulation Dimension (Focused Bandura): Main question; “How would you describe the moment just before entering the exam room and the first moment you stand before the jury?”. Example probe question; “What do you tell yourself to manage your nervousness? How does hearing the other candidates affect you?”

Qualitative Data Analysis: The descriptive analysis method was applied to the obtained data. The data were coded according to predetermined theoretical themes (cognitive, psychomotor, and affective); inductive coding was used for new situations that did not fit the theoretical framework but were frequently emphasized by the participants (Miles et al., 2014). The item generation matrix is presented in Table 2.

**Table 2:** Item generation matrix.

Student statement (Direct Quote)^1^	Emerging theme	Related theory	Generated draft item
*“…, I cannot discern the high pitches notes in between.”* (P2)	Auditory Discrimination	Gordon (Harmonic Audiation)	*Item 18: I am able to discriminate between the pitches during the polyphonic auditory test.*
*“… I cannot sustain the song until the end.”* (P9)	Physiological Control	Simpson (Mechanism)	*Item 28: I am able to maintain technically correct breath and vocal control, even under the stress of aptitude examinations.*
*“… thinking [this], I feel discouraged.”* (P1)	Social Comparison	Bandura (Vicarious Experience)	*Item 5: Hearing the performances of other candidates does not negatively affect my belief in my own success.*

^1^Details are given in Supplementary Table S1.

As a result, a broad draft pool of 47 items was created by combining theoretical points from the literature and statements derived from student opinions. The generated items are listed in Supplementary Table S1.

#### 2.3.2 Content validity

The draft form was presented to an expert panel of five expert in the fields of music education, measurement and evaluation, and psychological counseling. The expert panel was formed from academics and experts with an average of 15 years of professional experience, selected from the fields of Music Education (*n* = 2), Educational Psychology (*n* = 1), Measurement and Evaluation (*n* = 1), and Examiner/Teacher (*n* = 1), to ensure multidimensional content validity (Appendix A). Lynn (1986) states that a minimum of 3 experts are required for content validity studies and that 5 experts is a sufficient and reliable number for establishing content validity. Experts were asked to evaluate the items using the Davis Technique (1: Not Relevant - 4: Highly Relevant) (Davis, 1992). As a result of the evaluation, the Content Validity Index (S-CVI) was calculated as 0.86, which is above the threshold of 0.80 (Polit and Beck, 2006), thus concluding that the items have achieved content validity. In line with expert recommendations, 12 items were removed from the pool, and some technical terms were simplified (Supplementary Table S2).

#### 2.3.3 Face validity and pilot study

To test the comprehensibility of the 35-item draft form, whose content validity was ensured based on expert opinion, a cognitive interviewing study was conducted among the target candidate (16–18 age group). In this stage, the “Think Aloud” and “Verbal Probe” techniques suggested by Willis (2004) were used.

The study group consisted of 15 students selected from different grade levels (11th and 12th grade) and different instrument groups. In one-on-one interviews lasting ap-proximately 20 min each, students were asked to read the items aloud, and their meaning-making processes were explored through questions such as, “What does this item bring to mind?” and “How would you express this word in your words?”

As a result of the interviews, technical terms found in academic literature but potentially ambiguous for the target audience’s age level were identified. For instance, it was determined that students perceived the expression ‘playing clearly and distinctly’ more accurately than the term ‘articulation,’ and ‘the tone/mode of the melody’ more accurately than ‘tonality’. Five items that caused semantic confusion were rephrased, technical terms were simplified, and the scale’s instructions were adapted to suit students’ music aptitude exam psychology, making it ready for the pilot application (Supplementary Table S3). The 35 statements included in the draft scale are designed to be rated on a scale of 1 to 5 (1: Never, 5: Completely) to best reflect the level of competence in that situation.

### 2.4 Quantitative data analysis

In the statistical analysis of the quantitative data, JASP 0.95.4 was used for descriptive statistics, Exploratory Factor Analysis (EFA), Confirmatory Factor Analysis (CFA) and reliability calculations. The analysis process was carried out following a four-stage protocol:

#### 2.4.1 Data screening and assumption testing

Prior to analysis, the dataset (*N* = 2,165) was scanned for erroneous entries and missing data. The multivariate normality assumption and outliers were examined using the Mahalanobis distance criterion (*p* < 0.001); outliers exceeding the critical threshold (*χ*^2^) were removed from the dataset (Tabachnick and Fidell, 2019). The normal distribution of the data was checked using the criteria of skewness and kurtosis coefficients being within the ±1.5 range (George and Mallery, 2024). Following these processes, 2030 data points worth using in the next stage of quantitative data analysis were identified and randomly divided into two independent subsamples.

#### 2.4.2 Structural exploration: exploratory factor analysis

The first independent sub-sample (*n* = 997) was utilized for the Exploratory Factor Analysis (EFA) to uncover the latent structure of the scale. Prior to extraction, the suitability of the data for factor analysis was assessed; the Kaiser-Meyer-Olkin (KMO) measure of sampling adequacy was required to exceed the threshold of 0.80, and Bartlett’s Test of Sphericity needed to be statistically significant (*p* < 0.001) to confirm adequate correlation among the variables (Tabachnick and Fidell, 2019). To reveal the underlying dimensions, Principal Axis Factoring (PAF) was employed. PAF was specifically preferred over Principal Component Analysis (PCA) as it focuses exclusively on common variance and is methodologically more robust for identifying latent theoretical constructs (Costello and Osborne, 2005). Given the theoretical assumption that the cognitive, psychomotor, and affective dimensions comprising music aptitude are inherently interrelated, an oblique rotation method, Promax (*ϰ* = 4), was applied rather than orthogonal rotation. To rigorously determine the optimal number of factors to retain, a multicriteria approach was adopted: (a) the Kaiser criterion (Eigenvalues > 1), (b) visual inspection of the Scree Plot, and (c) Parallel Analysis results. During the iterative item reduction process, items were eliminated if their primary factor loading was below 0.40 or if they exhibited significant cross-loading (defined as a loading difference of less than 0.10 between their primary and secondary factor loadings; Hair et al., 2019).

#### 2.4.3 Structural validation: confirmatory factor analysis

The second sub-sample, consisting of 1,033 participants, was utilized for the Confirmatory Factor Analysis (CFA). In the literature, the number of participants per observed variable or item (N:p ratio) is considered a critical criterion in determining sample size. Bentler and Chou (1987) state that at least 5 participants per item (5:1 ratio) is sufficient for reliable parameter estimations. In this study, this ratio was calculated as approximately 49:1 (1,033/21) for the 21-item final form. Consequently, the sample size provides highly robust statistical power for model validation, far exceeding the acceptable limits defined by Tabachnick and Fidell (2019) and Hair et al. (2019).

To test the construct validity of the three-factor measurement model (Cognitive-Auditory, Psychomotor-Performance, and Affective Regulation) identified through EFA, a first-order CFA was specified. Given that the data substantially met the assumption of multivariate normality, the Maximum Likelihood (ML) estimation method was applied (Byrne, 2013). During model specification, the covariances among the latent fac-tors were freely estimated, and the variance of the first observed variable for each latent factor was fixed to 1 to establish scale metric (unit loading identification).

Consistent with current psychometric reporting standards (Brown, 2015; Kline, 2023), model fit was evaluated using a combination of absolute and comparative goodness-of-fit indices to avoid reliance on a single metric. The examined absolute indices included the *χ*^2^/df (Chi-square/degrees of freedom) ratio, RMSEA (Root Mean Square Error of Approximation), and SRMR (Standardized Root Mean Square Residual). Additionally, the CFI (Comparative Fit Index) and TLI (Tucker-Lewis Index) were investigated as comparative indices. The interpretation of these parameters strictly followed the combinatorial rule strategy proposed by Hu and Bentler (1999). The established reference ranges for acceptable fit indices (Browne et al., 1993; Marsh et al., 2004) are presented in Table 3.

**Table 3:** Fit indices.

Fit indices	Excellent fit	Acceptable fit	References
*χ^2^*/*df*	0 ≤ *χ^2^*/*df* ≤ 2	2 ≤ *χ^2^*/*df* ≤ 4	Kline (2023)
RMSEA	≤ 0.06	≤ 0.08	Hu and Bentler (1999)
SRMR	≤ 0.05	≤ 0.08	Byrne (2013)
CFI	≥ 0.95	≥ 0.90	Hu and Bentler (1999)
TLI	≥ 0.95	≥ 0.90	Bentler and Chou (1987)

#### 2.4.4 Evidence for reliability and validity

In the reliability analysis of the scale, in addition to the traditional Cronbach’s Alpha (*α*) coefficient, McDonald’s Omega (*ω*) and Composite Reliability (CR) coefficients—which provide more robust results as they are based on factor loadings—were reported. For convergent validity, the criteria established were an Average Variance Extracted (AVE) value above 0.50, a Composite Reliability (CR) value above 0.70, and the fulfillment of the CR > AVE condition (Fornell and Larcker, 1981). Discriminant validity was examined based on the criterion that the square root of the AVE values should be higher than the inter-factor correlations.

### 2.5 Concurrent validity

Although the construct validity analyses of the MASES were conducted using split subsamples, the criterion-related concurrent validity analyses were performed utilizing the entire merged dataset (*N* = 2030). This approach was strategically adopted to maximize statistical power and ensure the robustness of the parameter estimations (Cohen, 2013; Tabachnick and Fidell, 2019). Specifically, Pearson product–moment correlation coefficients were examined between the total scores of the finalized 21-item MASES and the scores derived from two established external criterion instruments: the SEQ-C and the SESRMA.

#### 2.5.1 Self-efficacy questionnaire for children

To assess domain-general self-efficacy, the SEQ-C, originally developed by Muris (2001) and adapted into Turkish by Çelikkaleli et al. (2006), was utilized. The SEQ-C is a 23-item, 5-point Likert-type instrument (1 = Not at all, 5 = Very well) designed to measure adolescents’ general competence beliefs. The scale encompasses three distinct sub-dimensions: Academic Self-Efficacy (8 items), Social Self-Efficacy (8 items), and Emotional Self-Efficacy (7 items). All items are positively scored, with cumulative scores ranging from 23 to 115, where higher scores reflect a greater level of generalized self-efficacy belief. Previous adaptation studies reported adequate internal consistency coefficients for the sub-dimensions, ranging between 0.64 and 0.71.

#### 2.5.2 Self-efficacy scale regarding musical ability

To evaluate domain-specific concurrent validity, the SESRMA, developed by Özmenteş and Özmenteş (2008), was administered. This 20-item, 5-point Likert-type instrument measures adolescents’ self-efficacy levels specifically concerning their musical aptitude. It captures self-efficacy beliefs shaped both by students’ personal self-assessments and by the perceived evaluations from their immediate social environment (e.g., family, peers, and teachers). The original scale development study confirmed a unidimensional (single-factor) structure and reported a highly robust internal consistency coefficient of *α* = 0.90.

### 2.6 Predictive validity

To determine the predictive validity and ecological robustness of MASES under realistic stress conditions, a live performance simulation was rigorously designed. A nested subsample of 50 candidates (*n* = 50, 100% 12th grade) was selected on a voluntary basis to participate in a high-stakes music aptitude test simulation. To accurately capture levels of expectancy self-efficacy, test-related anxiety, and emotional regulation, candidates completed MASES exactly 24 h prior to their scheduled performance. The evaluation panel consisted of a 5-member expert jury comprised of university academics (*n* = 3) and highly experienced music educators (*n* = 2). The jury independently assessed each candidate’s live performance (covering cognitive-auditory and psychomotor domains) using a standardized national aptitude assessment scale scored out of 100. Importantly, a rigorous blind protocol was implemented: jury members were completely unaware of the candidates’ MASES scores, and candidates were evaluated solely based on their real-time performance. The final empirical performance score for each candidate was calculated as the arithmetic mean of five independent expert evaluations. Finally, the predictive power of the scale was analyzed by calculating Pearson correlation coefficients between candidates’ pre-examination MASES scores (total and sub-dimensions) and the actual performance results given by the jury.

## 3 Results

### 3.1 Data screening and assumption testing

Prior to conducting the primary analyses, the raw dataset comprising 2,165 participants was subjected to rigorous screening to address missing data, identify outliers, and evaluate normality assumptions. To detect multivariate outliers, the Mahalanobis distance metric was calculated. Following the conservative criteria recommended by Tabachnick and Fidell (2019), 135 cases exceeding the critical chi-square threshold (*χ*^2^ = 66.62, *p* < 0.001, df = 35) were identified and subsequently excluded from the analysis. This process yielded a final, robust dataset of 2,030 participants for the subsequent factor analyses.

Normality tests conducted on the remaining dataset (*N* = 2030) showed that the items’ skewness (−1.01 to 0.71) and kurtosis (−1.10 to 0.99) values were within acceptable limits (±1.50). However, Item 5 (“I believe that those around me think I will succeed in the aptitude test”) was strategically removed from the item pool prior to factor extraction. This exclusion was justified both statistically—as its kurtosis value (1.83) exceeded the stringent univariate normality threshold adopted for this study—and theoretically, due to its peripheral contribution to the core construct of individual self-efficacy (George and Mallery, 2024).

Prior to factor extraction, a preliminary item analysis was conducted to evaluate the discrimination levels of the items in the pool. The Critical Ratio (CR) method, which compares the item scores of the upper 27% and lower 27% cohorts of the sample, con-firmed strong item discrimination with statistically significant t-values for all items (ranging from 3.94 to 13.71, *p* < 0.001) (Ebel, 1972). However, upon examining the Corrected Item-Total Correlation (CITC) values, nine items (i2, i8, i12, i16, i19, i26, i27, i28, i29; r range: 0.16 to 0.39) failed to meet the stringent discrimination threshold of 0.40 and were systematically eliminated (Nunnally and Bernstein, 1994). Following this reduction, the CITC discrimination coefficients of the remaining 25 items ranged from 0.42 to 0.64, and the preliminary internal consistency (*α*) of the scale increased from 0.90 to 0.92 (Supplementary Table S4).

To test construct validity and improve the generalizability of the model, the two-step validation approach was adopted (Anderson and Gerbing, 1988; Worthington and Whittaker, 2006). In this context, the screened dataset was randomly partitioned into two independent subsamples. The first subsample (*n* = 997) was utilized for Exploratory Factor Analysis (EFA) to uncover the latent factor structure of the remaining 25 items, while the second subsample (*n* = 1,033) was reserved for Confirmatory Factor Analysis (CFA) to validate the emergent structure.

### 3.2 Exploratory factor analysis

To determine the factor structure of the scale, which was reduced to 25 items, EFA was applied to the first subsample (*n* = 997) using the JASP 0.95.4 program. Principal Axis Factoring (PAF) was preferred as the inference method due to its effectiveness in revealing latent variables, and Promax (Oblique) was chosen as the rotation method because the factors are theoretically expected to be related (cognitive, psychomotor, and affective processes) (Costello and Osborne, 2005; Hair et al., 2019).

An iterative procedure was rigorously followed during the factor extraction process. The initial Principal Axis Factoring (PAF) conducted on the 25-item pool, corroborated by the Parallel Analysis results, indicated a clear three-factor latent structure. However, a meticulous examination of the pattern matrix revealed that four items (i1, i15, i20, i30) failed to meet the predetermined primary factor loading threshold of 0.40. In accordance with the methodological guidelines outlined by Hair et al. (2019), these items were systematically removed from the model. Notably, no items exhibited problematic cross-loadings (i.e., a loading difference of less than 0.10 across multiple factors) during this phase. Following these exclusions, the EFA was re-estimated, culminating in a robust and final structure comprising 21 items optimally aligned under three distinct theoretical dimensions.

**Figure 2:**
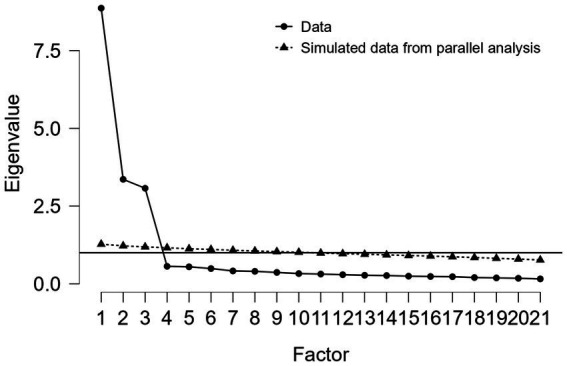
Scree plot of EFA.

The final EFA results obtained with 21 items showed that the data were perfectly suitable for factor analysis (KMO = 0.935; Bartlett’s Test *χ*^2^(210) = 17210.384, *p* < 0.001). Parallel analysis and the Scree Plot confirmed the three-factor structure, consistent with theoretical expectations (Figure 2). The factor loadings, common variances, and dimension assignments are shown in Table 4.

**Table 4:** Factor loadings and communalities from the EFA of the 21-item scale (*n* = 997).

Items	Factor 1	Factor 2	Factor 3	h^2^
i24	0.886			0.756
i35	0.865			0.728
i33	0.851			0.705
i32	0.844			0.735
i23	0.834			0.690
i34	0.832			0.694
i31	0.824			0.675
i22	0.809			0.726
i25	0.795			0.674
i13		0.880		0.728
i17		0.847		0.707
i10		0.844		0.731
i11		0.804		0.636
i18		0.792		0.629
i14		0.737		0.609
i21		0.715		0.536
i3			0.886	0.767
i7			0.829	0.704
i4			0.829	0.677
i6			0.782	0.640
i9			0.775	0.608
Eigenvalues	8.555	3.043	2.753	
% of Variance	40.7	14.5	13.1	
Cumulative %	40.7	55.2	68.3	68.3 (Total)
McDonald’s ω	0.956	0.929	0.912	0.921 (Total)
Cronbach’s α	0.955	0.928	0.911	0.926 (Total)

Applied rotation method is promax. Factor loadings < 0.40 are suppressed; h^2^ = communalities. Bartlett’s Test of Sphericity (*χ*^2^ = 17210.384; df = 210; *p* < 0.001) and KMO measure (KMO = 0.935).

According to Table 4, the factor loadings of all items are above the threshold value of 0.40, ranging from 0.715 to 0.886. All items on the scale showed sufficient common variance (range: 0.536–0.767). The scale resulted in a final 21-item structure with a clear three-factor structure explaining 68.3% of the total variance. Furthermore, Harman’s single-factor test was conducted to assess the potential threat of common method variance. The analysis revealed that a single unrotated factor explained 39.9% of the total variance. Given that this value is substantially below the established 50% threshold, it provides preliminary evidence that common method bias is not a pervasive issue in this dataset. However, as this test is primarily diagnostic, it should be noted that it does not clearly rule out all potential method effects.

**Figure 3:**
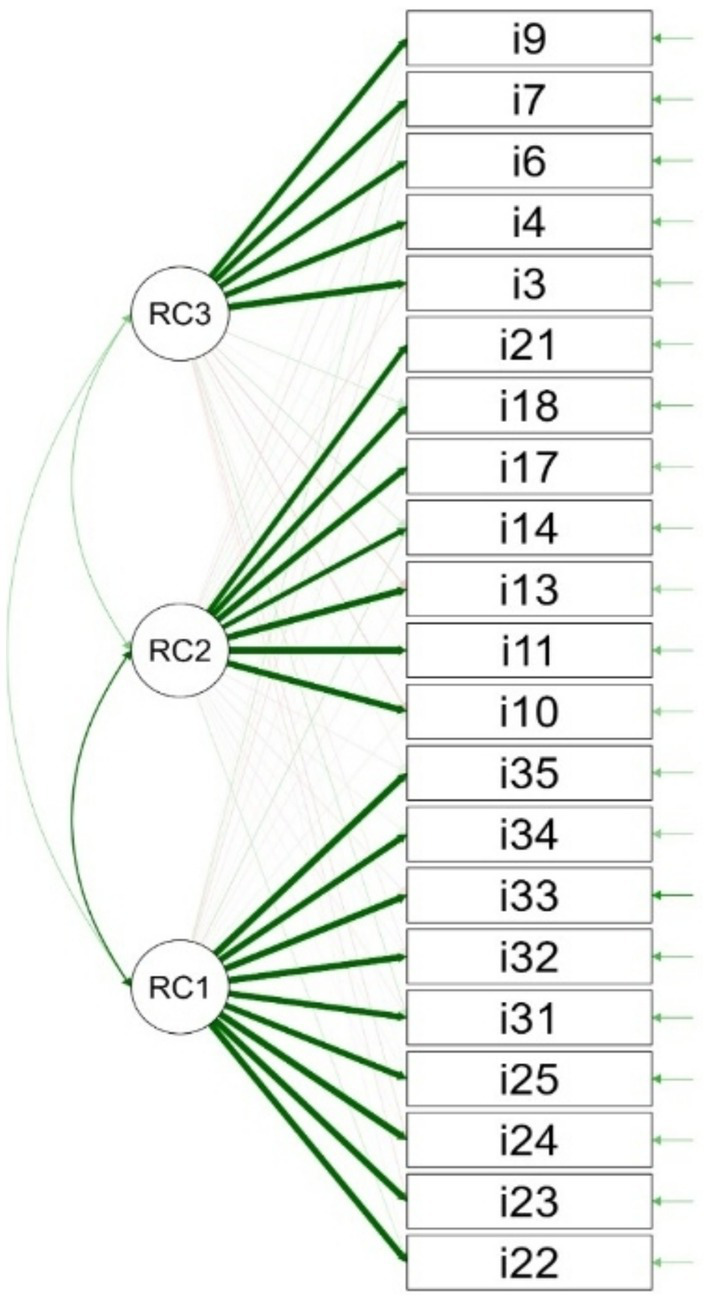
Path diagram of triadic model (MASES).

**Figure 4:**
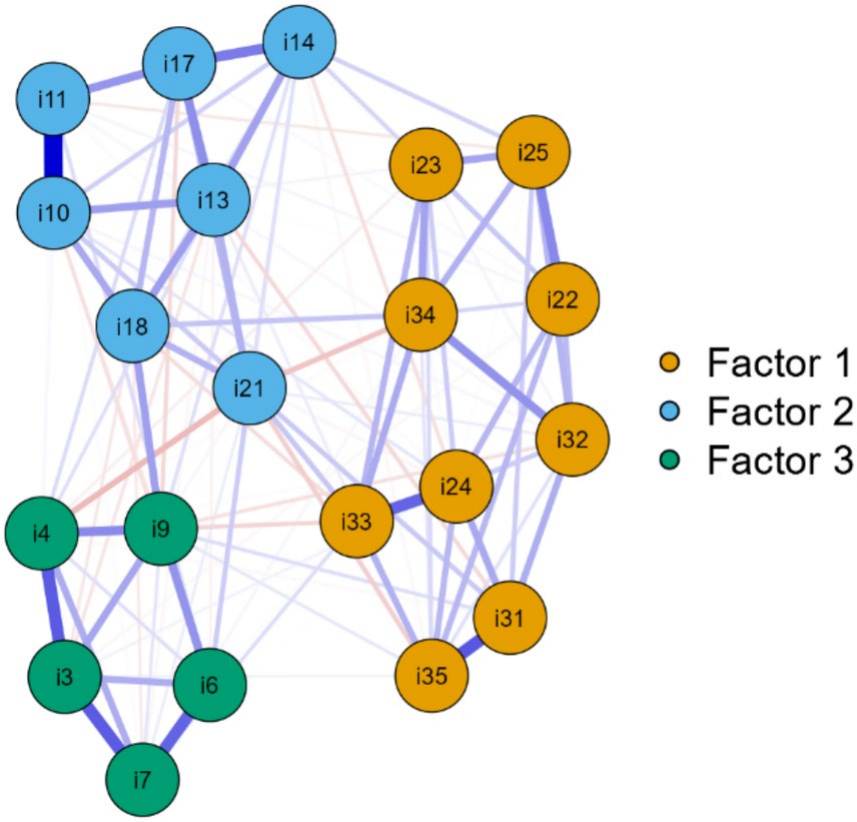
Network graphic of triadic model (MASES).

The resulting structure offers the best balance in terms of statistical fit and theoretical interpretability (Hayton et al., 2004; Stevens, 2012; Comrey and Lee, 2013; Hair et al., 2019; Tabachnick and Fidell, 2019). The factors were theoretically named; Factor 1 (Psycho-motor- Performance) consists of 9 items, Factor 2 (Cognitive-Auditory) consists of 7 items, and Factor 3 (Affective Regulation) consists of 5 items (Figure 3A). Cronbach’s *α* values are 0.955, 0.928, and 0.911, respectively, with an overall scale α value of 0.926. The path diagram and network graphic of the scale are shown in Figures 3, 4.

As visually represented in the network graphic (Figure 3B), the inter-item correlations within the latent factors of the final 21-item scale ranged between 0.06 and 0.77. These values remain well below the strict 0.90 threshold, thereby confirming the absence of multicollinearity and item redundancy.

### 3.3 Confirmatory factor analysis

Before conducting the Confirmatory Factor Analysis, the data distribution characteristics of the subsample (*n* = 1,033) used in the analysis were examined. The condition of multivariate normality, which is the fundamental assumption of the Maximum Likelihood (ML) estimation method, was checked. It was observed that the skewness and kurtosis values of the items ranged within the ±1.5 range (Tabachnick and Fidell, 2019) and Mardia’s multivariate normality coefficients were within acceptable limits for the analysis. Furthermore, based on the Mahalanobis distance criterion (*p* < 0.001), it was determined that there were no multivariate outliers in this subgroup that would bias the analysis (Figure 4).

To test the accuracy of the three-factor structure identified in the EFA phase, Confirmatory Factor Analysis (CFA) was performed on the second subsample (*n* = 1,033). The goodness-of-fit indices for the Confirmatory Factor Analysis are presented in Table 5.

**Table 5:** Confirmatory factor analysis fit indices.

Model	*χ^2^*	*df*	*χ^2^/df*	CFI	TLI	SRMR	RMSEA
Triadic	1179.818*	186	6.34	0.945	0.938	0.036	0.072**

*p = 0.001; **[90% CI (0.068–0.076)].

According to Table 5, the goodness-of-fit values of the model were observed, and the Chi-Square value was statistically significant (*χ*^2^ = 1179.818, df = 186, *p* = 0.001). However, since the sensitivity of the Chi-Square value to sample size is known (Kline, 2023), the *χ*^2^/df ratio was used to evaluate the model fit. The calculated ratio of 6.34 (*χ*^2^/df > 5) indicates that the model does not show a very good fit. Indicates that the model does not show a very good fit. Although this ratio slightly exceeds the traditional threshold of 5.0, it is well-documented in the psychometric literature that the chi-square statistic is hypersensitive to large sample sizes (e.g., N > 1,000), often leading to inflated values and the rejection of well-fitting models (Bollen, 2014; Brown, 2015; Hair et al., 2019; Kline, 2023). Consequently, evaluating the model fit through a combination of other robust absolute and incremental fit indices is highly recommended. When examining these supplementary fit indices, the three-factor measurement model demonstrated an acceptable fit to the empirical data: Comparative Fit Index (CFI) = 0.945, Tucker-Lewis Index (TLI) = 0.938, Root Mean Square Error of Approximation (RMSEA) = 0.072 [90% CI (0.068, 0.076)], and Standardized Root Mean Square Residual (SRMR) = 0.036. Specifically, the RMSEA value of 0.072 indicates an acceptable fit, and the incremental fit indices (CFI, TLI) suggest an overall adequate model (Browne et al., 1993; Hu and Bentler, 1999). Furthermore, an examination of the parameter estimates revealed that all items loaded strongly and significantly onto their respective latent constructs. The standardized factor loadings were highly robust, ranging from 0.81 to 0.87 for the Psychomotor-Performance factor, 0.73 to 0.85 for the Cognitive-Auditory factor, and 0.77 to 0.87 for the Affective Regulation factor (all *p* < 0.001). These results, achieved without requiring any post-hoc error covariance modifications, adequately support the structural validity of the triadic MASES model. Consequently, the acceptable structural fit indices and robust construct validity findings support Hypothesis 1 (H1), confirming the triadic nature of the scale.

### 3.4 Reliability and validity

To clearly establish the psychometric quality of the scale, comprehensive internal consistency, composite reliability, and convergent validity analyses were conducted. For the evaluation of internal reliability, both the traditional Cronbach’s Alpha (*α*) and McDonald’s Omega (*ω*) coefficients were utilized. The inclusion of ω is methodologically significant, as it provides a more robust estimation of reliability based on actual factor loadings without assuming strict tau-equivalence. To evaluate convergent validity, the Average Variance Extracted (AVE) and Composite Reliability (CR) metrics were rigorously investigated. The integrated reliability and convergent validity results are presented in Table 6.

**Table 6:** Reliability and convergent validity results.

Factor	*k*	*α*	ω	CR	AVE
*F_1_	9	0.956	0.957	0.955	0.710
*F_2_	7	0.934	0.935	0.928	0.675
*F_3_	5	0.907	0.908	0.912	0.665
Total	21	0.928	0.962		

α: Internal consistency coefficient; ω: Omega reliability coefficient; CR, Composite Reliability; AVE, Average Variance Extracted; F_1_, Psychomotor- Performance, F_2_, Cognitive-Auditory, F_3_, Affective Regulation. *All factor variances: *p* < 0.001.

In evaluating the MASES, we adopted a contemporary psychometric perspective, conceptualizing construct validity not as a singular statistical endpoint, but as a continuous process of accumulated evidence (Clark and Watson, 2019). While foundational metrics such as Average Variance Extracted (AVE) and Composite Reliability (CR) provide essential structural information, a comprehensive validation framework must also account for item content relevance, relationships with external criteria, non-redundancy with existing scales, stability across demographic subgroups, and the practical consequences of the scale’s deployment in high-stakes examination contexts. By synthesizing these diverse strands of evidence across multiple phases of this study, we aimed to robustly substantiate the utility and theoretical integrity of the MASES.

As detailed in Table 6, the *α* (0.907–0.956) and *ω* (0.908–0.957) values for all specific dimensions and the overall scale substantially exceeded the recommended 0.70 threshold, indicating an exceptionally high level of internal consistency (Hair et al., 2019). Regarding convergent validity, the AVE values for the respective factors—Factor 1 (0.710), Factor 2 (0.675), and Factor 3 (0.665)—robustly satisfied the ≥ 0.50 acceptable minimum threshold. Furthermore, the CR values for all latent constructs ranged from 0.912 to 0.955, significantly surpassing the 0.70 criterion and fulfilling the fundamental convergent validity assumption (CR > AVE). Given these rigorous metrics alongside highly significant factor loadings (*p* < 0.001), the convergent validity and internal reliability of the developed scale are conclusively verified.

Discriminant validity was evaluated using the conservative Fornell-Larcker criterion. According to this protocol, the square root of the AVE for each respective latent construct must be greater than its zero-order correlations with other constructs in the model. As detailed in the correlation matrix (Table 7), the square root of the AVE values on the diagonal (ranging from 0.815 to 0.843) were strictly higher than the off-diagonal inter-factor correlations (ranging from 0.207 to 0.466). These findings conclusively demonstrate that each dimension of the MASES shares more variance with its own specified indicators than with other constructs, thereby establishing robust discriminant validity. To provide a more robust and contemporary evaluation of discriminant validity, the Heterotrait-Monotrait (HTMT) ratio of correlations was also examined, as recommended in current psychometric literature. All HTMT values between the sub-dimensions of the MASES were strictly below the conservative threshold of 0.85 (maximum HTMT = 0.46). The fulfillment of both the Fornell-Larcker criterion and the HTMT threshold provides robust evidence that the three sub-dimensions of the scale represent theoretically distinct constructs.

**Table 7:** Discriminant validity (Fornell-Larcker Criterion) and inter-factor correlations.

Factors	1	2	3
1. Factor 1	**0.843**		
2. Factor 2	0.466*	**0.822**	
3. Factor 3	0.259*	0.207*	**0.815**

### 3.5 Concurrent validity

To test the concurrent validity of the developed MASES, the SEQ-C and the SESRMA were utilized as a criterion. When the normality distribution of the data was examined, it was observed that the skewness and kurtosis coefficients varied within the ±1.5 range. These values indicate that the data meet the assumption of normal distribution and that parametric tests are applicable (Kline, 2023; George and Mallery, 2024). The results of the reliability analysis of the criterion scales are presented in Table 8.

**Table 8:** The results of the reliability analysis of the criterion scales.

Scale	*k*	Cronbach’s *α*	McDonald’s *ω*
Academic self-efficacy	8	0.847	0.848
Social self-efficacy	8	0.829	0.830
Emotional self-efficacy	7	0.873	0.874
the SEQ-C	23	0.929	0.930
the SESRMA	20	0.920	0.914

According to Table 8, the Cronbach Alpha (α) and McDonald’s Omega (ω) coefficients range from 0.829 to 0.930. The fact that the obtained coefficients are well above the 0.70 confidence threshold accepted in the literature (Nunnally, 1978; Hair et al., 2019) indicates that the criterion scales have a high level of internal consistency within the scope of this study. The results of the Pearson correlation analysis between the MASES and criterion scales are presented in Table 9.

**Table 9:** The results of the Pearson correlation analysis between the MASES and criterion scales.

Variables	Academic SE	Social SE	Emotional SE	the SEQ-C	the SESRMA
Factor 1	0.368**	0.472**	0.438**	0.483**	0.682**
Factor 2	0.372**	0.384**	0.397**	0.436**	0.564**
Factor 3	0.400**	0.364**	0.382**	0.432**	0.388**
the MASES	0.503**	0.560**	0.550**	0.609**	0.768**

*N* = 2030; **p < 0.001. Factor 1: Psychomotor- Performance, Factor 2: Cognitive-Auditory, Factor 3: Affective Regulation; SE, Self-Efficacy.

Table 9 shows that there is a statistically significant, positive, and moderate-to-high correlation between the MASES total score and the criterion scales total scores (0.609 and 0.768). In the literature, correlation values between 0.30 and 0.70 are considered ideal, as they indicate that the developed scale theoretically overlaps with the criterion scale while measures a different construct (Büyüköztürk, 2007; Cohen, 2013).

When examined at the sub-dimension level, it was observed that all sub-dimensions of the MASES (F1, F2, F3) exhibited positive and significant correlations with the sub-dimensions of the SEQ-C (Academic, Social, Emotional) ranging from 0.364 to 0.472. Notably, the strong relationship between the MASES total score and the Social Self-Efficacy (*r* = 0.560) and Emotional Self-Efficacy (*r* = 0.550) dimensions demonstrates that musical aptitude self-efficacy is not only a technical skill but is also closely linked to social and emotional self-efficacy beliefs. These coefficients provide strong evidence that criterion-related validity has been established in addition to the construct validity of the MASES.

Crucially, when examining the domain-specific criterion, the MASES exhibited a strong and highly significant positive correlation with the established unidimensional SESRMA (*r* = 0.77, *p* < 0.001). This robust convergence provides definitive empirical evidence that the newly developed MASES accurately captures the core construct of music-related self-efficacy. Importantly, while the magnitude of this correlation demonstrates strong concurrent validity, it does not approach unity (*r* < 0.90). This indicates that the triadic structure of the MASES (Psychomotor, Cognitive, and Affective dimensions) captures unique and nuanced variance regarding musical aptitude self-efficacy that is not entirely encompassed by the existing unidimensional instrument, thereby further justifying its development.

These findings collectively provide significantly empirical evidence for the criterion-related validity of the MASES. Collectively, these findings provide significantly empirical evidence for the concurrent validity of the MASES across both general and specific domains, thereby fully supporting Hypothesis 2 (H2).

### 3.6 Predictive validity

To evaluate the predictive validity of the MASES, Pearson product–moment correlations were calculated between the candidates’ pre-performance MASES scores (collected 24 h prior), their general academic grade point average (GPA), and the objective performance scores rigorously awarded by the 5-member expert jury (*n* = 50). The empirical analysis revealed a highly significant and strong positive correlation between the MASES total score and the actual jury performance score [*r* = 0.784, 95% CI (0.684, 0.872), *p* < 0.001]. This significant finding indicates that the candidates’ anticipatory self-efficacy successfully predicted their objective musical aptitude performance under high-stakes conditions (Table 10).

**Table 10:** Pearson correlations among MASES scores, GPA, and jury performance score.

Variables	Factor 1	Factor 2	Factor 3	the MASES	GPA
Jury PS (%95 CI)	0.415* (0.154–0.621)	0.936** (0.890–0.963)	0.068 (−0.214–0.340)	0.784** (0.648–0.872)	0.422* (0.162–0.627)
GPA (%95 CI)	0.032 (−0.249–0.308)	0.451** (0.197–0.648)	0.235 (−0.047–0.482)	0.334* (0.062–0.561)	–

*N* = 50; *p < 0.05, **p < 0.001. Factor 1: Psychomotor- Performance, Factor 2: Cognitive-Auditory, Factor 3: Affective Regulation; PS: Performance Score; GPA: Academic Grade Point Average.

At the specific sub-dimensional level, the Cognitive-Auditory dimension exhibited a near-perfect predictive relationship with the jury scores [*r* = 0.936, 95% CI (0.890, 0.963), *p* < 0.001], while the Psychomotor-Performance dimension showed a moderate, yet highly significant correlation [*r* = 0.415, 95% CI (0.154, 0.621), *p* < 0.01]. Interestingly, the Affective Regulation dimension did not significantly predict the objective technical jury score [*r* = 0.068, 95% CI (−0.214, 0.340), *p* = 0.638]. As critically noted, the near-perfect correlation between the cognitive-auditory dimension and objective jury performance must be interpreted with caution. This magnitude suggests potential construct overlap and criterion contamination, as items measuring students’ self-efficacy beliefs regarding rhythm, intonation, and auditory memory closely mirror the precise technical parameters in the expert jury’s evaluation rubric. Furthermore, the divergence of Affective Regulation aligns theoretically with the nature of the standardized assessment rubric, which predominantly prioritized cognitive-auditory processing (e.g., pitch and rhythm reproduction) and technical psychomotor execution over psychological stage presence, thus underscoring the multidimensional discriminant sensitivity of the MASES.

To formally evaluate the incremental validity of the MASES in predicting live musical performance, a hierarchical multiple regression analysis was conducted exclusively on the predictive validity subsample (*n* = 50). The objective Jury Performance Score was designated as the dependent variable. In Step 1, academic achievement (GPA) was entered into the model to control for general academic ability. In Step 2, the three MASES dimensions were entered. The results (see Table 11) indicated that in the first step, GPA significantly accounted for 17.8% of the variance in jury scores (*R*^2^ = 0.178, *p* = 0.002). The inclusion of the MASES dimensions in Step 2 resulted in a substantial and significant increase in the explained variance, accounting for an additional 75.3% of the variance in jury performance (Δ*R*^2^ = 0.753, *p* < 0.001). The full model explained a total of 93.1% of the variance in objective evaluation outcomes (*R*^2^ = 0.931, *p* < 0.001). Examination of the individual standardized coefficients in Step 2 revealed that when controlling for general academic achievement, Psychomotor-Performance emerged as the strongest positive predictor (*β* = 0.89, *p* < 0.001), followed by Cognitive-Auditory self-efficacy (*β* = 0.21, *p* < 0.001). Affective Regulation demonstrated a small negative predictive value (*β* = −0.09, *p* = 0.042). Ultimately, this robust empirical evidence supports Hypothesis 3 (H3), demonstrating that domain-specific anticipatory self-efficacy significantly predicts actual musical performance beyond traditional generalized academic metrics.

**Table 11:** Hierarchical multiple regression analysis predicting jury performance score.

Model/Predictor	B	SE	*β*	*t*	*p*	95% CI [LL, UL]
Step 1						
(Constant)	10.32	15.16		0.68	0.499	[−20.16, 40.81]
GPA	0.61	0.19	0.42	3.22	0.002	[0.230, 0.993]
Model 1 summary	*R*^2^ = 0.18		*F* = 10.38	*p =* 0.002		
Step 2						
(Constant)	6.54	4.98		1.31	0.195	[−3.481, 16.56]
GPA	0.05	0.07	0.03	0.73	0.469	[−0.083, 0.178]
Cognitive-auditory	3.15	0.61	0.21	5.13	< 0.001	[1.911, 4.384]
Psychomotor-performance	13.78	0.70	0.89	19.6	< 0.001	[12.36, 15.19]
Affective regulation	−1.40	0.67	−0.09	−2.09	0.042	[−2.750, −0.051]
Model 2 summary	Δ*R*^2^ = 0.75			*p* < 0.001		
Total model summary	*R*^2^ = 0.93		*F* = 151.20	*p* < 0.001		

N, 50. B, Unstandardized coefficient; SE, Standard error; β, Standardized coefficient; CI, Confidence Interval; LL, Lower Limit; UL, Upper Limit.

## 4 Discussion

The main objective of this study is to develop a valid and reliable measurement tool that can measure the musical self-efficacy perceptions of adolescents (16–18 years old) preparing for music aptitude tests or exams. The research findings revealed that the developed scale (the MASES) exhibits a Triadic Model structure consisting of Cognitive-Auditory, Psychomotor-Performance, and Affective Regulation, and has strong psychometric evidence.

A review of the literature reveals that valuable measurement tools on self-efficacy and motivation in the musical field have been developed by McPherson and McCormick (2006) and Ritchie and Williamon (2012). However, these tools generally focus on general musical performance and do not address the specific components required by aptitude tests, such as ‘auditory coding (dictation)’, ‘instant deciphering’, and ‘jury stress’. The developed scale (the MASES) differs from the existing literature in that it is the first assessment tool to base the multidimensional structure of aptitude tests on the Triadic Model, which includes Cognitive, Psychomotor, and Affective aspects.

### 4.1 Validation of the factor structure (triadic model)

In complete support of Hypothesis 1 (H1), the primary finding of the current study establishes that anticipatory self-efficacy regarding musical aptitude is not a monolithic, unidimensional construct, but rather a complex psychological phenomenon built upon three complementary pillars. The “Cognitive-Auditory” dimension, which emerged robustly in the exploratory and confirmatory analyses, represents the candidate’s belief in their musical perception, tonal memory, and theoretical decoding. Conversely, the “Psychomotor-Performance” dimension reflects confidence in the physical and technical execution of those musical skills. This triadic differentiation aligns seamlessly with existing literature, which dictates that high-level musical performance demands the intricate synchronization of cognitive processing and motor execution (McPherson and McCormick, 2006; Hallam, 2010; Barros et al., 2022).

Crucially, the emergence of the “Affective Regulation” dimension as a distinct third pillar confirms the valuable role of emotional management and stage anxiety during high-stakes aptitude assessments. Given that performance anxiety—particularly prevalent during adolescence—can debilitate cognitive and motor functions and directly undermine self-efficacy beliefs (Kenny, 2011; Aufegger et al., 2017; Borges et al., 2026), incorporating this dimension significantly broadens the scale’s ecological validity regarding examination psychology. The emergence of the ‘Emotional Regulation’ dimension largely aligns with recent educational psychology research in the literature. For instance, Trigueros et al. (2020) demonstrated that effectively managing test anxiety and academic stress is a fundamental component of psychological resilience in educational settings. By quantifying this emotional management, the MASES operationalizes the adaptive coping strategies necessary to mitigate debilitating performance anxiety. By effectively quantifying how adolescents cope with these severe educational stressors, the MASES contributes vital empirical evidence to the broader literature on promoting emotional health, preventing burnout, and fostering psychological well-being within highly competitive academic settings. Ultimately, the acceptable structural fit indices obtained in the CFA phase (RMSEA = 0.072, CFI = 0.945) provide significantly empirical evidence that this triadic model effectively captures the multifaceted nature of musical aptitude self-efficacy, thereby surpassing the limitations of traditional unidimensional measures.

### 4.2 Reliability and robust measurement accuracy

The psychometric evaluation of the MASES demonstrated exceptional reliability and structural accuracy. Internal consistency was rigorously assessed utilizing both the traditional Cronbach’s *α* and the contemporary McDonald’s *ω* coefficients, yielding highly robust values (ranging from 0.907 to 0.962) across all sub-dimensions and the total scale. The integration of the ω coefficient is particularly significant in modern scale development; unlike α, ω does not rely on the restrictive assumption of strict tau-equivalence (i.e., equal factor loadings across all items), thereby providing a more precise and unbiased estimation of true reliability (Hayes and Coutts, 2020).

Beyond internal consistency, the measurement accuracy of the MASES was clearly solidified through robust convergent and discriminant validity metrics. All latent factors produced Average Variance Extracted (AVE) values well above the 0.50 threshold and Composite Reliability (CR) values exceeding 0.90, confirming that the constructs capture a substantial portion of the variance from their specific indicators. Furthermore, the successful satisfaction of the conservative Fornell-Larcker criterion proved that the triadic dimensions (Cognitive, Psychomotor, and Affective) function as distinct, non-overlapping theoretical pillars.

Collectively, these advanced psychometric properties indicate that the MASES is a highly sensitive instrument capable of producing stable, low-error measurements. Consequently, it stands as a useful and reliable assessment tool for educators and researchers in evaluating psychological readiness and profiling individual student trajectories prior to high-stakes musical aptitude assessments.

### 4.3 Dual-criterion concurrent validity: general and domain-specific alignment

A significantly contribution of the current study, which fully supports Hypothesis 2 (H2), is the empirical demonstration of the scale’s concurrent validity through a dual-criterion approach. When evaluated against the domain-general Self-Efficacy Questionnaire for Children (SEQ-C), the MASES exhibited a strong positive correlation (*r* = 0.609). This finding seamlessly aligns with Bandura’s (1997) theoretical assertion that although self-efficacy is domain-specific, it is inherently fueled by a broader foundation of general competence beliefs. It is particularly noteworthy that the “Affective Regulation” dimension of the MASES showed robust associations with the “Social” and “Emotional” competency dimensions of the generalized scale. This statistically supports the premise that high-stakes musical aptitude tests are not merely evaluations of technical competence, but rather complex processes requiring substantial socio-emotional resilience. This significant overlap between affective regulation and generalized emotional self-efficacy is theoretically supported by Freire et al. (2016), who highlighted that adaptive coping strategies are inextricably linked to students’ overall psychological well-being. Furthermore, Vizoso et al. (2019) emphasize that possessing robust coping mechanisms and positive competence beliefs are critical protective factors for preventing academic burnout. Thus, the MASES effectively captures the socio-emotional buffers required for sustainable talent development. Consequently, it can be inferred that adolescents who possess high musical self-efficacy also tend to exhibit greater confidence in their generalized life skills, and vice versa.

Furthermore, the ecological validity of the MASES was clearly anchored by its relationship with the domain-specific Self-Efficacy Scale Regarding Musical Ability (SESRMA). The MASES demonstrated a highly significant convergence with this established unidimensional measure (*r* = 0.768). This robust association confirms that the newly developed scale accurately targets the core psychological construct of musical aptitude. However, it is theoretically crucial that the magnitude of this correlation, while strong, did not reach statistical redundancy (*r* < 0.90). This threshold indicates that the triadic structure of the MASES captures unique, multidimensional variance—particularly regarding intricate psychomotor and affective nuances—that older, unidimensional instruments fail to encompass. Ultimately, the MASES successfully bridges the psychometric gap between broad psychological resilience and highly specialized musical competence.

### 4.4 Predictive validity and ecological robustness in live performance

The most methodologically innovative aspect of the current study is the empirical validation of the MASES through a high-stakes live performance simulation. The exceptionally high inter-rater reliability among the independent expert jury was confirmed using a two-way mixed-effects model, absolute agreement, average measures [ICC = 0.955, 95% CI (0.916, 0.974)], indicating exceptional inter-rater agreement for the performance metric. Against this robust criterion, the predictive power of the MASES yielded striking results. Confirming Hypothesis 3 (H3), the strong correlation (*r* = 0.784) between candidates’ pre-examination self-efficacy and their actual live performance provides definitive evidence that the MASES is not merely a theoretical construct, but a highly accurate predictor of real-world behavioral outcomes.

A particularly significantly finding emerged regarding the incremental validity of the scale. While generalized academic achievement (GPA) was only moderately associated with musical performance (*r* = 0.422), the MASES demonstrated substantially superior predictive capacity. This empirically highlights the inadequacy of general academic metrics in identifying musical aptitude and underscores the necessity of employing domain-specific diagnostic tools, such as the MASES, in talent selection processes (Orejudo et al., 2021). The strong predictive power of the scale under live stress conditions echoes the critical findings of Morales-Rodríguez (2021) regarding the absolute necessity of robust coping strategies when students confront severe environmental and academic stressors. Similarly, Asikainen et al. (2020) established that a student’s ability to manage study-related exhaustion and stress directly dictates their objective academic achievement. In this context, the MASES proves to be an ecologically valid tool that bridges internal coping capacity with external, high-stakes performance. The performance impact of affective regulation programs can occur not only directly but also indirectly by managing systemic anxiety. Indeed, current literature (Vasiou et al., 2025) shows that high emotional intelligence and emotion regulation practices centrally increase academic performance (GPA) in higher education by reducing technological test anxiety (worry/concern). The functioning of Affective Regulation in our models is consistent with this finding, and it has begun to function as a buffering mechanism for healthy anxiety under evaluator stress in music aptitude tests.

At the sub-dimensional level, the near-perfect predictive power of the Cognitive-Auditory dimension (*r* = 0.936) reveals that adolescents possess valuable metacognitive awareness regarding their specific aural capacities (e.g., tonal memory and pitch discrimination). Conversely, the lack of a significant predictive relationship between the Affective Regulation dimension and the objective jury score (*r* = 0.068) requires a more balanced interpretation. While this may indicate that the standardized jury rubric primarily evaluated exact technical execution rather than internal psychological states, it is equally plausible that the Affective Regulation dimension simply has limited direct predictive value for this specific objective criterion. In highly technical assessments, affective coping mechanisms might operate strictly as an internal buffering system against debilitating anxiety, rather than directly translating into measurable technical points. Nevertheless, this divergence substantiates the discriminant validity of the scale’s sub-dimensions, confirming that internal emotional resilience is a distinct construct from technical auditory capacity. Ultimately, the MASES proves to be a highly ecologically valid instrument capable of mapping the intricate psychological landscape of a candidate before they ever step onto the stage. However, this exceptionally high coefficient must be interpreted critically. Such a near-perfect correlation raises the possibility of construct overlap and criterion contamination, as the scale’s specific cognitive-auditory items closely mirror the precise technical parameters evaluated by the jury rubric. Additionally, the specific context of the live-performance simulation may have introduced restricted variance. Therefore, while the predictive capacity is evident, the magnitude of this relationship may be partially inflated by these methodological overlaps.

### 4.5 Limitations and recommendations for future research

Despite its strong psychometric properties and methodological advantages, the current study has several limitations. Firstly, the sample size is geographically and contextually limited to adolescent musicians in Türkiye preparing for national or international exams. Therefore, future research should prioritize external validation in independent samples and cross-cultural adaptation studies to validate the MASES’s tripartite structure across diverse populations and different musical contexts. Furthermore, from a structural perspective, while the primary fit indices (e.g., CFI, TLI, and RMSEA) demonstrated acceptable model fit, it is important to acknowledge that the *X*^2^/df ratio (6.34) exceeded strict traditional thresholds. While this inflation is largely attributable to the well-documented sensitivity of the chi-square statistic to large sample sizes (CFA *n* = 1,033), relying predominantly on conventional fit thresholds has inherent limitations. Therefore, to achieve a more nuanced understanding of this construct, future psychometric research should advance beyond the traditional confirmatory approach by testing alternative structural frameworks. Specifically, employing higher-order models, bifactor modeling, or Exploratory Structural Equation Modeling (ESEM) could provide deeper insights into the complex, multidimensional nature of musical anticipatory self-efficacy.

Furthermore, while this study successfully demonstrated short-term predictive validity through a rigorous live performance simulation, the sample size for this specific phase was relatively small and voluntary (*n* = 50). This introduces the possibility of self-selection bias, as candidates who were more confident in their musical abilities may have been more likely to participate. Additionally, the performance assessment was a simulation rather than a high-stakes exam with actual university admission outcomes. Future longitudinal studies are strongly recommended to track candidates’ MASES scores against actual admission results and subsequent academic success in professional music education.

Methodologically, there is a potential predictor-criterion overlap to consider. The exceptionally high correlation between the Cognitive-Auditory dimension and the jury score may be partially inflated because the scale items closely mirror the technical parameters of the expert evaluation rubric. Moreover, this initial validation study did not comprehensively assess the measurement invariance of the MASES; therefore, future research should examine its configural, metric, and scalar invariance across different demographic variables such as gender and age. Additionally, although preliminary diagnostic tests indicated that common method bias was not a primary driver of the variance, future studies should employ procedural remedies and advanced statistical controls (e.g., a common latent factor marker) to completely isolate potential method variance.

Furthermore, moving beyond traditional psychometrics, future research should explore whether automated or computationally assisted detection methodologies can successfully identify cases with more difficult-to-manage profiles of performance anxiety or abnormally low self-efficacy (Mastrothanasis et al., 2023). Implementing such advanced methodologies should ultimately aim to provide targeted, early pedagogical support and psychological intervention for vulnerable candidates, rather than serving as a mechanism for the automatic selection or rejection of students.

Finally, a key recommendation to advance construct validity is to further investigate the Affective Regulation dimension. Because this particular dimension captures the psychological stress management aspect of anticipatory self-efficacy rather than technical application, future researchers are encouraged to rigorously test its convergent and discriminant validity against established clinical performance anxiety tools such as the Kenny Music Performance Anxiety Inventory (KMPAI). Such comparative studies will further illuminate the complex theoretical interplay between music self-efficacy, debilitating stage anxiety, and objective performance outcomes.

## 5 Conclusions and future directions

### 5.1 Conclusion

The current study successfully developed and psychometrically validated the Musical Aptitude Self-Efficacy Scale (MASES), a novel, 21-item instrument designed to precisely measure the anticipatory self-efficacy beliefs of adolescents preparing for high-stakes music aptitude examinations. Moving decisively beyond traditional unidimensional approaches, the MASES established a robust triadic structural model comprising Cognitive-Auditory, Psychomotor-Performance, and Affective Regulation dimensions. This multidimensional framework was rigorously cross-validated across a large-scale, diverse adolescent sample, demonstrating acceptable internal consistency, construct validity, and structural fit.

The scale further exhibited strong dual-criterion concurrent validity by aligning theoretically with both generalized socio-emotional resilience and domain-specific musical self-efficacy. Through a rigorous live performance simulation evaluated by an expert jury, candidates’ pre-examination MASES scores strongly predicted their objective technical performance. Crucially, the scale demonstrated substantial incremental validity over traditional generalized academic metrics, indicating that domain-specific anticipatory self-efficacy is a fundamentally superior predictor of actual musical aptitude.

In conclusion, the current empirical findings suggest that the MASES is a highly promising, ecologically valid, and robust psychometric tool. However, it is premature to deploy it as a fully mature diagnostic tool for high-stakes admission committees or formal selection procedures. To reach that level of practical utility, further rigorous checks are required, including the establishment of standardized norms, cut-off score validation, fairness testing, comprehensive measurement invariance across diverse demographic groups, and cross-cultural validation in independent samples. Ultimately, the MASES provides music educators and researchers with a valuable psychological lens to identify specific self-efficacy profiles and design targeted pedagogical interventions to foster more resilient musical talent.

### 5.2 Practical implications and future directions

Based on the robust empirical findings of the current study, the following actionable recommendations are provided for educational practitioners, audition committees, and future researchers.

#### 5.2.1 Pedagogical implications for educators and institutions

Diagnostic Assessment and Targeted Intervention: Music educators and psychological counselors at schools and preparatory academies can utilize the MASES as a proactive diagnostic tool. By identifying specific “at-risk” psychological profiles early in the training process—for instance, a student exhibiting high “Psychomotor-Performance” but critically low “Affective Regulation”—educators can prioritize targeted interventions, such as stress-inoculation training and frequent performance simulations, before the actual exam. Fostering this affective resilience is a critical step in preventing student burnout, protecting adolescent mental health (aligning with SDG 3: Good Health and Well-being), and ensuring a sustainable, supportive educational ecosystem where talent is nurtured rather than lost to anxiety (aligning with SDG 4: Quality Education).

Individualized Career Counseling: School guidance services can leverage the multi-dimensional scores of the scale to facilitate realistic career orientations. Students displaying persistently low self-efficacy across all dimensions, despite pedagogical support, can be guided in restructuring their academic expectations and exploring alternative goal settings.

Supplementary Non-Cognitive Metrics for Juries: Aptitude test committees and conservatory audition juries are encouraged to consider candidates’ MASES profiles as complementary, non-cognitive data during the qualitative evaluation phases (e.g., the interview stage). Understanding a candidate’s anticipatory belief in their own potential can provide invaluable context beyond their immediate, stress-induced technical performance.

#### 5.2.2 Methodological recommendations for future research

Macro-Level Predictive Validity: While this study validated predictive power through a rigorous live simulation, future research should investigate the logistic predictive validity of the MASES on actual national placement outcomes (e.g., official pass/fail admission status).

Temporal Fluctuation (Longitudinal Tracking): To map the developmental trajectory of musical self-efficacy, researchers should conduct repeated-measures longitudinal de-signs, tracking candidates’ MASES scores at the onset of preparation, mid-term, and exactly on the day of the official aptitude test.

Global Standardization and Clinical Comparisons: To transcend the Turkish examination context, future studies must prioritize the cross-cultural adaptation of the MASES for international music assessment boards (e.g., ABRSM, Trinity College London). Furthermore, rigorously comparing the “Affective Regulation” dimension with clinical performance anxiety inventories (such as the K-MPAI) will critically advance the theoretical understanding of the dynamic balance between anticipatory confidence and debilitating stage fright.

## Data Availability

The original contributions presented in the study are included in the article/Supplementary material, further inquiries can be directed to the corresponding author/s.
