# Supplementary material for: Anticipatory self-efficacy predicts live musical performance: development and validation of the Music Aptitude Self-Efficacy Scale
Source: Front Psychol. 2026 Jun 19;17:1869088. doi: 10.3389/fpsyg.2026.1869088 (PMC13328190; doi:10.3389/fpsyg.2026.1869088)
Supplement: Supplementary file 1 [file Supplementary_file_1.DOCX]

Supplementary Material

**Table S1.** Item Generation Matrix^1^

| **Student Statement (Direct Quote)** | **Emerging Theme (Code)** | **Relevant Theory** | **Generated Draft Item** |
| --- | --- | --- | --- |
| *"When I'm dictating, I panic if I miss a note; it's like I'm losing everything."* (P1) | Cognitive Disconnection / Panic | **Gordon** (Melodic Audiation) | *Item 16: I have difficulty memorizing melodies during auditory exams.* |
| *"I hear a sound from the piano, but when I try to reproduce that sound in my head with my voice, the sound falters, I become out of tune."* (P3) | Tonal Memory and Singing | **Gordon** (Tonal Audiation) | *Item 14:* *I rely on melodic memory during melodic repetition tests.* |
| *"During rhythm repetition, the teacher taps the table, and just as I'm about to do exactly the same, I forget the beginning of the rhythm."* (P8) | Rhythmic Short-Term Memory | **Gordon** (Rhythmic Audiation) | *Item 15: I can memorize rhythmic patterns during rhythm repetition tests.* |
| *"When they ask about chords, they all play them at once, so I only hear the noise. I cannot discern the high pitches notes in between." "* (P2) | Auditory Discrimination | **Gordon** (Harmonic Audiation) | *Item 18: I am able to discriminate between the pitches during the polyphonic auditory test.* |
| *"I'm scared the sight-reading piece will have too many sharps."* (P7) | Perception of Technical Inadequacy | Simpson  (Psikomotor) | *Item 25: The high density of modifier signs (sharps/flats) in the sight-reading piece does not affect performance.* |
| *"* *"When I'm playing the piece in the exam, if I get stuck at a certain point, I get all flustered and freeze up."* (P6) | Technical Continuity / Fluency | **Simpson** (Adaptation) | *Item 34: Even if I play a wrong note during instrument exams, I can keep going without stopping.* |
| *"When I get excited, I run out of breath, my voice trembles, I cannot sustain the song until the end." "* (P9) | Physiological Control (Breathing/Muscles) | **Simpson** (Mechanism) | *Item 28: I am able to maintain technically correct breath and vocal control, even under the stress of aptitude examinations.* |
| *"When they put the sight-reading piece in front of me, if the notes come too fast, my fingers can't keep up"* (P4) | Demonstrating Complex Skills | **Simpson** (Complex Overt Response) | *Item 23: Even if the tempo of the sight-reading piece is fast, I can technically perform it* |
| *"When I wait at the door and hear someone playing really well coming from inside, I think, I feel discouraged."* (P1) | Social Comparison / Indirect Experiences | **Bandura** (Vicarious Experience) | *Item 5: Hearing the performances of other candidates does not negatively affect my belief in my own success.* |
| *"When the jury stares at me so intensely, I forget what I know; it feels like they want to eliminate me."* (P7) | Threat of Social Evaluation | **Bandura** (Social Persuasion / Context) | *Item 30: I can focus on my performance regardless of the judges' attitudes.* |
| *"* *As the exam day approaches, I'm getting stomach pains, and my hands are sweating."* (P5) | Physiological and Emotional Conditions | **Bandura** (Physiological States) | *Item 3: I can manage the physical tension (sweating, trembling, etc.) I experience before aptitude tests* |
| *"If the jury gives me a stern look during the exam, my hands and feet will tremble, and I won't be able to play."*(P5) | Social Evaluation Anxiety | **Bandura** (Physiological States) | *Item 2: I can control my attention and thoughts in front of the jury.* |

^1^ This table provides examples of the process.
