# Supplementary material for: Anticipatory self-efficacy predicts live musical performance: development and validation of the Music Aptitude Self-Efficacy Scale
Source: Front Psychol. 2026 Jun 19;17:1869088. doi: 10.3389/fpsyg.2026.1869088 (PMC13328190; doi:10.3389/fpsyg.2026.1869088)
Supplement: Supplementary file 2 [file Supplementary_file_2.DOCX]

Supplementary Material

**Table S2.** Expert Rating Form and Content Validity Indices

| **Item No** | **Expert 1** | **Expert 2** | **Expert 3** | **Expert 4** | **Expert 5** | **Number of Agreements**  **(3 and 4 scores)** | **I-CVI (Item-Content Validity Index)** | **Decision** |
| --- | --- | --- | --- | --- | --- | --- | --- | --- |
| **i1** | 4 | 4 | 3 | 4 | 4 | 5 | **1.00** | **ACCEPTED** |
| **i2** | 3 | 4 | 4 | 4 | 3 | 5 | **1.00** | **ACCEPTED** |
| **i3** | 4 | 2 | 4 | 4 | 4 | 4 | **0.80** | **ACCEPTED** |
| **i4** | 4 | 2 | 4 | 3 | 4 | 4 | **0.80** | **ACCEPTED** |
| **i5** | 3 | 4 | 4 | 4 | 2 | 4 | **0.80** | **ACCEPTED** |
| **i6** | 4 | 4 | 4 | 4 | 4 | 5 | **1.00** | **ACCEPTED** |
| **i7** | 4 | 3 | 2 | 4 | 4 | 4 | **0.80** | **ACCEPTED** |
| **i8** | 4 | 4 | 4 | 4 | 3 | 5 | **1.00** | **ACCEPTED** |
| **i9** | 2 | 4 | 4 | 3 | 4 | 4 | **0.80** | **ACCEPTED** |
| **i10** | 4 | 4 | 4 | 4 | 4 | 5 | **1.00** | **ACCEPTED** |
| **i11** | 3 | 4 | 4 | 2 | 4 | 4 | **0.80** | **ACCEPTED** |
| **i12** | 4 | 2 | 4 | 4 | 3 | 4 | **0.80** | **ACCEPTED** |
| **i13** | 4 | 4 | 3 | 4 | 1 | 4 | **0.80** | **ACCEPTED** |
| **i14** | 4 | 4 | 4 | 3 | 4 | 5 | **1.00** | **ACCEPTED** |
| **i15** | 4 | 1 | 4 | 4 | 4 | 4 | **0.80** | **ACCEPTED** |
| **i16** | 3 | 4 | 4 | 4 | 4 | 5 | **1.00** | **ACCEPTED** |
| **i17** | 4 | 4 | 1 | 4 | 3 | 4 | **0.80** | **ACCEPTED** |
| **i18** | 4 | 4 | 4 | 4 | 4 | 5 | **1.00** | **ACCEPTED** |
| **i19** | 4 | 4 | 4 | 1 | 4 | 4 | **0.80** | **ACCEPTED** |
| **i20** | 2 | 4 | 3 | 4 | 4 | 4 | **0.80** | **ACCEPTED** |
| **i21** | 4 | 4 | 4 | 4 | 4 | 5 | **1.00** | **ACCEPTED** |
| **i22** | 4 | 4 | 4 | 4 | 2 | 4 | **0.80** | **ACCEPTED** |
| **i23** | 3 | 3 | 4 | 4 | 4 | 5 | **1.00** | **ACCEPTED** |
| **i24** | 4 | 2 | 4 | 4 | 3 | 4 | **0.80** | **ACCEPTED** |
| **i25** | 2 | 4 | 4 | 4 | 4 | 4 | **0.80** | **ACCEPTED** |
| **i26** | 4 | 4 | 4 | 3 | 4 | 5 | **1.00** | **ACCEPTED** |
| **i27** | 4 | 4 | 1 | 4 | 4 | 4 | **0.80** | **ACCEPTED** |
| **i28** | 3 | 4 | 4 | 2 | 4 | 4 | **0.80** | **ACCEPTED** |
| **i29** | 4 | 4 | 4 | 4 | 2 | 4 | **0.80** | **ACCEPTED** |
| **i30** | 4 | 4 | 4 | 4 | 2 | 4 | **0.80** | **ACCEPTED** |
| **i31** | 1 | 4 | 4 | 4 | 3 | 4 | **0.80** | **ACCEPTED** |
| **i32** | 3 | 4 | 4 | 4 | 4 | 5 | **1.00** | **ACCEPTED** |
| **i33** | 4 | 3 | 4 | 4 | 4 | 5 | **1.00** | **ACCEPTED** |
| **i34** | 4 | 4 | 2 | 4 | 4 | 4 | **0.80** | **ACCEPTED** |
| **i35** | 4 | 4 | 4 | 3 | 2 | 4 | **0.80** | **ACCEPTED** |
| **i36** | 2 | 4 | 2 | 3 | 4 | 3 | **0.60** | **ELIMINATED** |
| **i37** | 1 | 2 | 4 | 3 | 4 | 3 | **0.60** | **ELIMINATED** |
| **i38** | 2 | 3 | 2 | 4 | 1 | 2 | **0.40** | **ELIMINATED** |
| **i39** | 3 | 2 | 1 | 4 | 4 | 3 | **0.60** | **ELIMINATED** |
| **i40** | 2 | 1 | 4 | 3 | 2 | 2 | **0.40** | **ELIMINATED** |
| **i41** | 4 | 4 | 2 | 2 | 4 | 3 | **0.60** | **ELIMINATED** |
| **i42** | 4 | 2 | 3 | 1 | 4 | 3 | **0.60** | **ELIMINATED** |
| **i43** | 2 | 2 | 4 | 3 | 4 | 3 | **0.60** | **ELIMINATED** |
| **i44** | 2 | 4 | 4 | 1 | 3 | 3 | **0.60** | **ELIMINATED** |
| **i45** | 3 | 2 | 4 | 4 | 1 | 3 | **0.60** | **ELIMINATED** |
| **i46** | 1 | 2 | 4 | 3 | 2 | 2 | **0.40** | **ELIMINATED** |
| **i47** | 2 | 1 | 3 | 4 | 4 | 3 | **0.60** | **ELIMINATED** |
| **Sum** |  |  |  |  |  |  | **Mean** |  |
| **S-CVI** |  |  |  |  |  |  | **0.86** |  |

**Mathematical Verification (For Editor's Review)**

**Initial Number of Items:** 47

**Eliminated Items (I-CVI < .80):** 12 Adet

- **Eliminated***:* i36 - i47.

**Number of Remaining Items:** 35

**Distribution of Remaining Items:**

- Number of items with complete agreement (I-CVI = 1.00): **10**
- Number of items with high agreement (I-CVI = 0.80): **25**

**Calculation of Scale Content Validity Index (S-CVI/Ave):**

- Total I-CVI Score = (10 x 1.00) + (25 x 0.80) = 10 + 20 = 30
- S-CVI = 30 / 35 = 0.8571
- **Reported:** **.86**

**Expert Scoring Key (Davis Technique):**

- 1: Not Relevant
- 2: Somewhat Relevant
- 3: Quite Relevant
- 4: Highly Relevant

Note: While calculating the I-CVI, the 3 and 4 scores were coded as agreement (1), and the 1 and 2 scores were coded as disagreement (0).
