# Supplementary material for: Anticipatory self-efficacy predicts live musical performance: development and validation of the Music Aptitude Self-Efficacy Scale
Source: Front Psychol. 2026 Jun 19;17:1869088. doi: 10.3389/fpsyg.2026.1869088 (PMC13328190; doi:10.3389/fpsyg.2026.1869088)
Supplement: Supplementary file 3 [file Supplementary_file_3.DOCX]

Supplementary Material

**Table S3.** Summary of Item Revisions Based on Cognitive Interviews

| Madde No | Relevant Factor | Original Draft Statement  (After Expert Approval) | Student Feedback (Problem) | Revised Final Statement |
| --- | --- | --- | --- | --- |
| i2 | *Affective Regulation* | *"* *I can control my* ***cognitive*** *processes in front of the jury."* | *"* *I don't know what cognitive means."* (P2) | *"I can control my* ***attention and thoughts*** *in front of the jury."* |
| i12 | *Cognitive-Auditory* | *"* *I can retain the melody's* ***tonality*** *in my mind during the dictation test."* | *"When you say tonality, do you mean the mode or the pitch of the voice?"* (P9) | *"I can retain the* ***key (mode)*** *of the melody in my mind without losing it during the dictation test."* |
| i29 | *Affective Regulation* | *"I maintain my emotional* ***regulation*** *while performing in front of a jury."* | *"I don't know what regulation means. Does it mean controlling my excitement?" (Abstract concept not appropriate for developmental/age level)* (P7) | *"I can control my* ***excitement/anxiety*** *while performing in front of the jury."* |
| i30 | *Affective Regulation* | *"I don't panic when the* ***sight-reading*** *piece comes up in the exam."* | (No problem) *"Sight-reading means playing a piece we've never seen before, right then and there, I know that."* (P11) | *No changes were made.* |
| i31 | *Psychomotor- Performance* | *""I can fully adhere to the rules of* ***articulation*** *while performing the piece."* | *"Teacher, what does articulation mean? Does it mean playing with prominent teeth, or playing clearly and distinctly? I'm not entirely sure."* (P4) | *"I can perform the pieces accurately and clearly in instrumental exams."* |

Note: Following cognitive interviews (N=15), academic terms unsuitable for the cognitive and age levels of the students were identified. Based on data obtained via verbal probing techniques (Willis, 2005), 5 items were rephrased using the target audience's terminology.
