# Supplementary material for: Anticipatory self-efficacy predicts live musical performance: development and validation of the Music Aptitude Self-Efficacy Scale
Source: Front Psychol. 2026 Jun 19;17:1869088. doi: 10.3389/fpsyg.2026.1869088 (PMC13328190; doi:10.3389/fpsyg.2026.1869088)
Supplement: Supplementary file 4 [file Supplementary_file_4.DOCX]

Supplementary Material

**Table S4.** Draft Scale

| ITEMS | | FINAL SITUATION | |
| --- | --- | --- | --- |
| i1 | I believe I can learn the things I need to know while preparing for the aptitude tests. | Low load in EFA | **DELETED** |
| i2 | I can control my attention and thoughts in front of the jury. | Item analysis | **DELETED** |
| i3 | **I can manage the physical tension (sweating, trembling, etc.) I experience before aptitude tests** | **Factor 3 (Affective Regulation Competence)** | |
| i4 | **I believe I am ready for the aptitude tests.** | **Factor 3 (Affective Regulation Competence)** | |
| i5 | Hearing the performances of other candidates does not negatively affect my belief in my own success. | +1,5- 1,5 | **DELETED** |
| i6 | **I believe the musical training that I received will be sufficient to pass the talent exams.** | **Factor 3 (Affective Regulation Competence)** | |
| i7 | **If I make a mistake on the exam, I can focus on the next stage without dwelling on that mistake.** | **Factor 3 (Affective Regulation Competence)** | |
| i8 | I need extra lessons to prepare for the musical performance stage of the talent exams. | Item analysis | **DELETED** |
| i9 | **Even if I don't feel physically well when I enter the exam room, I believe I can still focus on the exam.** | **Factor 3 (Affective Regulation Competence)** | |
| i10 | **I think I can understand the measure of the melody while writing dictation in the melody dictation exam.** | **Factor 2 (Cognitive-Auditory Competence)** | |
| i11 | **I think I can understand the tonality of the melody while writing dictation in the melody dictation exam.** | **Factor 2 (Cognitive-Auditory Competence)** | |
| i12 | I can retain the key (mode) of the melody in my mind without losing it during the dictation test | Item analysis | **DELETED** |
| i13 | **I think I can understand the rhythm of the melody while writing dictation in the melody dictation exam.** | **Factor 2 (Cognitive-Auditory Competence)** | |
| i14 | **I rely on melodic memory during melodic repetition tests** | **Factor 2 (Cognitive-Auditory Competence)** | |
| i15 | Even if the tempo of the sight-reading piece is fast, I can technically perform it. | Low load in EFA1 | **DELETED** |
| i16 | I have difficulty memorizing melodies during auditory exams. | Item analysis | **DELETED** |
| i17 | **I can memorize rhythmic patterns during rhythm repetition tests.** | **Factor 2 (Cognitive-Auditory Competence)** | |
| i18 | **I am able to discriminate between the pitches during the polyphonic auditory test.** | **Factor 2 (Cognitive-Auditory Competence)** | |
| i19 | I believe I can clearly reproduce the intervals I hear in multi-tone hearing tests. | Item analysis | **DELETED** |
| i20 | In solfège sight-reading exams, the vocal range of the sight-reading technique does not affect performance. | Low load in EFA | **DELETED** |
| i21 | **I believe my knowledge of intervals will be sufficient for solfège sight-reading exams.** | **Factor 2 (Cognitive-Auditory Competence)** | |
| i22 | **In solfège sight-reading exams, I can correctly vocalize the intervals in the melody.** | **Factor 1 (Psychomotor- Performance Competence)** | |
| i23 | **In solfège sight-reading exams, I can correctly vocalize the rhythmic structure of the melody.** | **Factor 1 (Psychomotor- Performance Competence)** | |
| i24 | **I can perform the piece musically in the instrument exam.** | **Factor 1 (Psychomotor- Performance Competence)** | |
| i25 | **In rhythmic reading tests, I can correctly vocalize the rhythmic patterns in the piece.** | **Factor 1 (Psychomotor- Performance Competence)** | |
| i26 | I believe I will be able to perform the pieces accurately and clearly in the singing exams. | Item analysis | **DELETED** |
| i27 | I believe I will be able to articulate and perform the pieces correctly in the singing exams. | Item analysis | **DELETED** |
| i28 | I am able to maintain technically correct breath and vocal control, even under the stress of aptitude examinations. | Item analysis | **DELETED** |
| i29 | I can control my excitement/anxiety while performing in front of the jury. | Item analysis | **DELETED** |
| i30 | I don't panic when the sight-reading piece comes up in the exam. | Low load in EFA | **DELETED** |
| i31 | **I can perform the pieces accurately and clearly in instrumental exams.** | **Factor 1 (Psychomotor- Performance Competence)** | |
| i32 | **I can perform the pieces accurately and clearly in singing exams.** | **Factor 1 (Psychomotor- Performance Competence)** | |
| i33 | **I can demonstrate my technical skills in the instrument sight-reading exam.** | **Factor 1 (Psychomotor- Performance Competence)** | |
| i34 | **Even if I play a wrong note during instrument exams, I can keep going without stopping.** | **Factor 1 (Psychomotor- Performance Competence)** | |
| i35 | **Even if I sing a wrong note in the singing test, I can keep going without stopping.** | **Factor 1 (Psychomotor- Performance Competence)** | |
