# Supplementary material for: Anticipatory self-efficacy predicts live musical performance: development and validation of the Music Aptitude Self-Efficacy Scale
Source: Front Psychol. 2026 Jun 19;17:1869088. doi: 10.3389/fpsyg.2026.1869088 (PMC13328190; doi:10.3389/fpsyg.2026.1869088)
Supplement: Supplementary file 5 [file Supplementary_file_5.DOCX]

Supplementary Material

**Appendix A: Expert Rating Form and Content Validity Indices**

**Expert Panel Composition**

1. **Expert 1 (Field Specialist - Theory/Auditory):**
   - Department: Faculty Member in Music Education (Associate Professor)
   - Role: Supervising Gordon's "Auditation" items (dictation, sight-reading, memory).
   - Reason: Because they are the academic authority who best understands the technical challenges of auditory examinations.
2. **Expert 2 (Field Specialist - Instrument Training):**
   - Department: Faculty Member in Music Education / Instrument Training (Dr. Lecturer)
   - Role: To supervise Simpson's "Psychomotor" (Execution, sight-reading, technique) aspects.
   - Reason: To verify instrument mastery and technical terms (articulation, nuance, etc.) regarding stage performance.
3. **Expert 3 (Education Physiology):**
   - Department: Faculty Member in Education Physiology (Prof. Dr.)
   - Role: To review Bandura's "Affective" (Anxiety, self-efficacy) items.
   - Reason: To discern whether the items truly measure "self-efficacy" or merely "motivation," and to check their suitability for adolescent psychology.
4. **Expert 4 (Methodology - Measurement and Evaluation):**
   - Department: Faculty Member in Education/ Measurement and Evaluation (Associate Professor)
   - Role: To review the structural quality of items.
   - Reason: To verify whether the item construction is technically correct.
5. **Expert 5 (Field Specialist - Practitioner):**
   - Department: Fine Arts High School Music Teacher (Experienced in aptitude test juries).
   - Role: To check the suitability of the items for the "real exam environment".
   - Reason: To examine the scale based on their experiences in the field.
