# Supplementary material for: Anticipatory self-efficacy predicts live musical performance: development and validation of the Music Aptitude Self-Efficacy Scale
Source: Front Psychol. 2026 Jun 19;17:1869088. doi: 10.3389/fpsyg.2026.1869088 (PMC13328190; doi:10.3389/fpsyg.2026.1869088)
Supplement: Supplementary file 6 [file Supplementary_file_6.DOCX]

Supplementary Material

Appendix B - The Music Aptitude Self-Efficacy Scale (MASES)

"Please rate each statement below from 1 to 5 (1: Never, 5: Completely) by choosing the number that best reflects your level of competence in that situation."

| **Statements** | **Rating 1-5** | **FACTORS** |
| --- | --- | --- |
| 1. I can manage the physical tension (sweating, trembling, etc.) I experience before aptitude tests |  | Factor 3 (Affective Regulation Competence) |
| 1. I believe I am ready for the aptitude tests. |  | Factor 3 (Affective Regulation Competence) |
| 1. I believe the musical training that I received will be sufficient to pass the talent exams. |  | Factor 3 (Affective Regulation Competence) |
| 1. If I make a mistake on the exam, I can focus on the next stage without dwelling on that mistake. |  | Factor 3 (Affective Regulation Competence) |
| 1. Even if I don't feel physically well when I enter the exam room, I believe I can still focus on the exam. |  | Factor 3 (Affective Regulation Competence) |
| 1. I think I can understand the measure of the melody while writing dictation in the melody dictation exam. |  | Factor 2 (Cognitive-Auditory Competence) |
| 1. I think I can understand the tonality of the melody while writing dictation in the melody dictation exam. |  | Factor 2 (Cognitive-Auditory Competence) |
| 1. I think I can understand the rhythm of the melody while writing dictation in the melody dictation exam. |  | Factor 2 (Cognitive-Auditory Competence) |
| 1. I rely on melodic memory during melodic repetition tests |  | Factor 2 (Cognitive-Auditory Competence) |
| 1. I can memorize rhythmic patterns during rhythm repetition tests. |  | Factor 2 (Cognitive-Auditory Competence) |
| 1. I am able to discriminate between the pitches during the polyphonic auditory test. |  | Factor 2 (Cognitive-Auditory Competence) |
| 1. I believe my knowledge of intervals will be sufficient for solfège sight-reading exams. |  | Factor 2 (Cognitive-Auditory Competence) |
| 1. In solfège sight-reading exams, I can correctly vocalize the intervals in the melody. |  | Factor 1 (Psychomotor- Performance Competence) |
| 1. In solfège sight-reading exams, I can correctly vocalize the rhythmic structure of the melody. |  | Factor 1 (Psychomotor- Performance Competence) |
| 1. I can perform the piece musically in the instrument exam. |  | Factor 1 (Psychomotor- Performance Competence) |
| 1. In rhythmic reading tests, I can correctly vocalize the rhythmic patterns in the piece. |  | Factor 1 (Psychomotor- Performance Competence) |
| 1. I can perform the pieces accurately and clearly in instrumental exams. |  | Factor 1 (Psychomotor- Performance Competence) |
| 1. I can perform the pieces accurately and clearly in singing exams. |  | Factor 1 (Psychomotor- Performance Competence) |
| 1. I can demonstrate my technical skills in the instrument sight-reading exam. |  | Factor 1 (Psychomotor- Performance Competence) |
| 1. Even if I play a wrong note during instrument exams, I can keep going without stopping. |  | Factor 1 (Psychomotor- Performance Competence) |
| 1. Even if I sing a wrong note in the singing test, I can keep going without stopping. |  | Factor 1 (Psychomotor- Performance Competence) |
