# Supplementary material for: Anticipatory self-efficacy predicts live musical performance: development and validation of the Music Aptitude Self-Efficacy Scale
Source: Front Psychol. 2026 Jun 19;17:1869088. doi: 10.3389/fpsyg.2026.1869088 (PMC13328190; doi:10.3389/fpsyg.2026.1869088)
Supplement: Supplementary file 7 [file Supplementary_file_7.DOCX]

Supplementary Material

**Appendix C.** Standardized National Music Aptitude Assessment Protocol (Live Performance Simulation)

**1. Overview, Sample, and Assessment Environment**

The live performance assessment was meticulously designed to simulate the high-stakes standardized national aptitude exams utilized for admission to fine arts high schools and state conservatories. The simulation was conducted with a nested subsample of 50 voluntary 12th-grade candidates. To ensure acoustic clarity, physiological safety, and the prevention of external distractions, the assessment environment was completely isolated. Each candidate was evaluated individually in a continuous session lasting approximately 10 to 15 minutes.

**2. Assessment Timeline and Strict Blind Protocol**

To accurately capture the psychological state of the candidates—specifically anticipatory self-efficacy, pre-performance anxiety, and emotional regulation—all 50 candidates completed the MASES exactly 24 hours prior to their scheduled live performance.

The live performance evaluation was conducted by a 5-member expert jury panel, comprising university academics (n = 3) and highly experienced music educators (n = 2). A rigorous blind evaluation protocol was enforced: jury members were entirely unaware of the candidates' prior MASES scores, ensuring that grading was strictly based on real-time performance. The final empirical performance score for each candidate was calculated as the arithmetic mean of the five independent expert evaluations out of 100 points.

**3. Standardized Evaluation Rubric (Total: 100 Points)**

The assessment rubric was explicitly structured to evaluate the candidates' cognitive-auditory processing and psychomotor execution capabilities, directly mirroring the theoretical latent dimensions of the MASES.

**PART I: Cognitive-Auditory Domain (Musical Hearing and Memory) – Maximum 70 Points**

This section measures the candidate's cognitive capacity to perceive, decode, retain, and accurately reproduce musical stimuli (pitch, rhythm, and melody) under examination stress.

- **A. Pitch Discrimination / Sound Recognition (Total: 30 Points)**
  - *Single Pitch:* Reproducing 1 sound played on the piano vocally (2 trials, 1 point each = **2 Points**).
  - *Two-Part Harmonics:* Reproducing 2 simultaneous sounds (3 trials, 2 points each = **6 Points**).
  - *Three-Part Chords:* Reproducing 3 simultaneous sounds (4 trials, 3 points each = **12 Points**).
  - *Four-Part Chords:* Reproducing 4 simultaneous sounds (2 trials, 5 points each = **10 Points**).
- **B. Rhythm Memory and Reproduction (Total: 20 Points)**
  - Reproducing two distinct rhythmic patterns (tapped by the examiner) by clapping or tapping.
  - *Scoring Criteria:* Accuracy of subdivisions, tempo maintenance, and cognitive recall (2 patterns, 10 points each).
- **C. Melodic Memory and Reproduction (Total: 20 Points)**
  - Reproducing two distinct melodic phrases (played on the piano, tonal and atonal variations) vocally with accurate intonation.
  - *Scoring Criteria:* Pitch accuracy, tonal center retention, and melodic contour (2 melodies, 10 points each).

**PART II: Psychomotor-Performance Domain (Vocal/Instrumental Execution) – Maximum 30 Points**

This section measures the candidate's psychomotor coordination, physical execution, and physiological control during real-time live performance.

- **A. Vocal or Instrumental Execution (15 Points)**
  - The candidate performs a prepared piece (vocal or instrumental) of their choice.
  - *Scoring Criteria:* Breath control (diaphragmatic support), vocal/instrumental resonance, diction/articulation, and precise psychomotor control of the vocal cords or gross/fine motor skills.
- **B. Physical Coordination and Rhythmic Expression (15 Points)**
  - Observation of the candidate's physical posture, hand-eye coordination (instrumentalists), and embodied rhythmic expression during the performance.
  - *Scoring Criteria:* Freedom from debilitating physical/performance tension, synchronization of motor skills, and overall stage presence.

**4. Inter-Rater Reliability of the Jury**

To evaluate the consistency and absolute agreement among the five independent expert jury members, the Intraclass Correlation Coefficient (ICC) was calculated. Utilizing a two-way random-effects model based on average-rating and absolute-agreement (ICC2k), the analysis yielded an exceptionally high reliability coefficient of ICC = .955 (95% CI [.933, .972], p < .001). According to the established guidelines by Koo and Li (2016), this value indicates "excellent" inter-rater reliability. This near-perfect agreement confirms that the standardized assessment rubric was applied with extreme consistency across all 50 candidates, thereby providing a highly robust and objective empirical performance metric for the predictive validity analysis.
